# Supplementary material for: Hepatitis B virus X protein (HBx)-mediated immune modulation and prognostic model development in hepatocellular carcinoma
Source: PLoS One. 2025 Jun 27;20(6):e0325363. doi: 10.1371/journal.pone.0325363 (PMC12204523; doi:10.1371/journal.pone.0325363)
Supplement: S1 Fig — A PPI network of HBx-related DEGs. B The number of interactions and interaction strength between different celltypes in HBV-related HCC tissues. C The interaction strength of MIF signaling pathway between different celltypes in HBV-related HCC tissues. D Signaling from malignant cells to other cells in the MIF signaling pathway. A thicker line indicates a higher interaction strength or a greater number of interactions. (PDF) [file pone.0325363.s001.pdf]

Supporting information

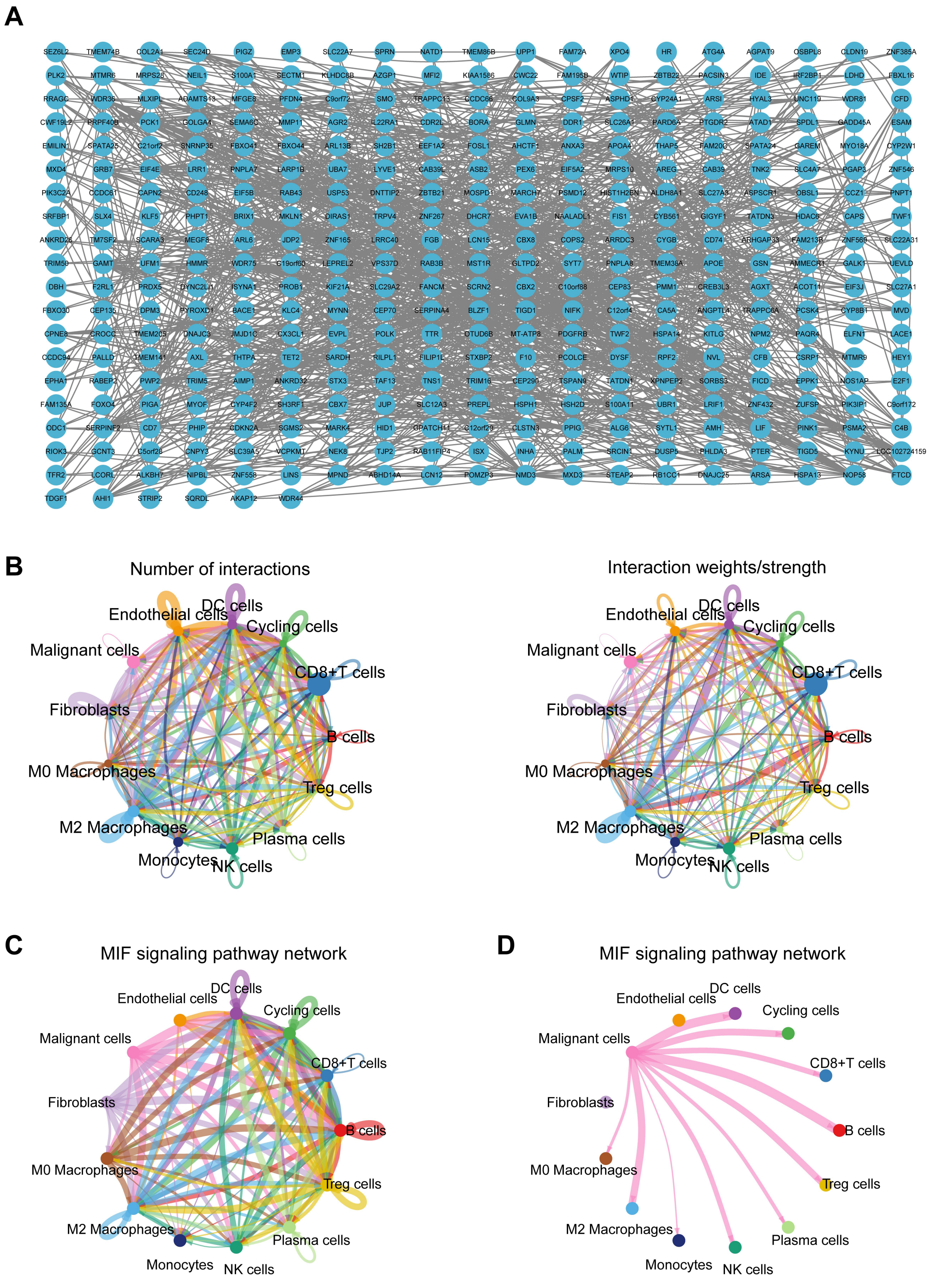

S1 Fig. Characterization of HBx-Related DEGs and MIF Signaling Interactions in HBV-Related HCC.

A PPI network of HBx-related DEGs. **B** The number of interactions and interaction strength between different celltypes in HBV-related HCC tissues. **C** The interaction strength of MIF signaling pathway between different celltypes in HBV-related HCC tissues. **D** Signaling from malignant cells to other cells in the MIF signaling pathway. A thicker line indicates a higher interaction strength or a greater number of interactions.
