# Supplementary material for: Hepatitis B virus X protein (HBx)-mediated immune modulation and prognostic model development in hepatocellular carcinoma
Source: PLoS One. 2025 Jun 27;20(6):e0325363. doi: 10.1371/journal.pone.0325363 (PMC12204523; doi:10.1371/journal.pone.0325363)
Supplement: S2 Fig — (PDF) [file pone.0325363.s002.pdf]

Supporting information

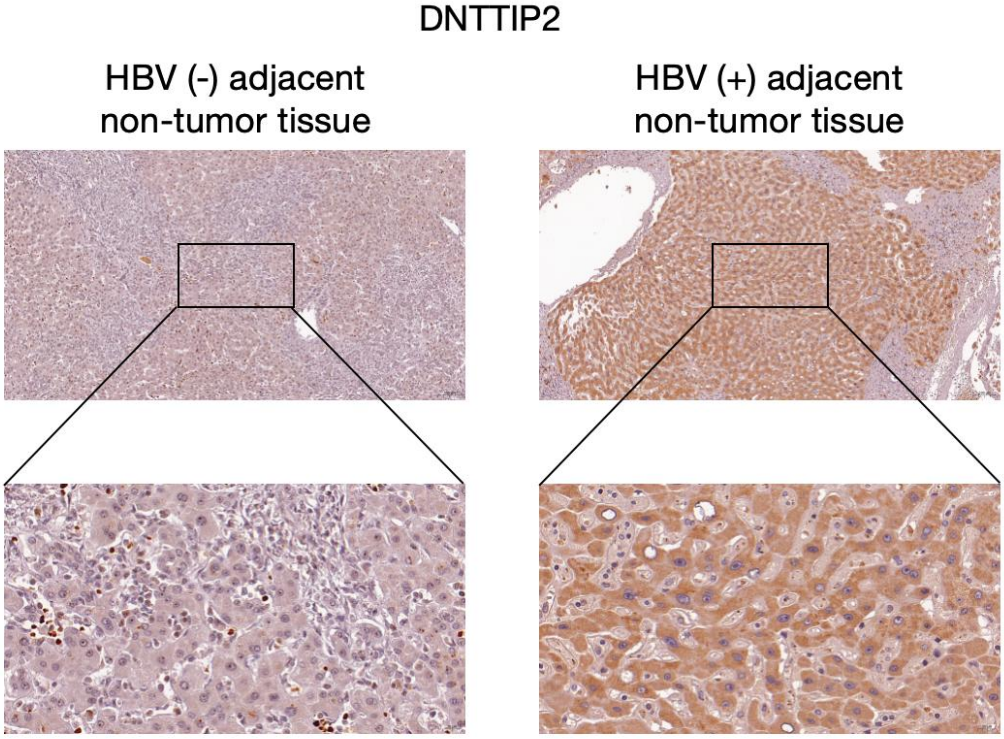

S2 Fig. Representative images of H&E staining and IHC staining of DNTTIP2 protein in HBV-negative and HBV-positive adjacent non-tumor tissues.
