# Supplementary figures and images for: Hepatitis B virus X protein (HBx)-mediated immune modulation and prognostic model development in hepatocellular carcinoma
Source: PLoS One. 2025 Jun 27;20(6):e0325363. doi: 10.1371/journal.pone.0325363 (PMC12204523; doi:10.1371/journal.pone.0325363)

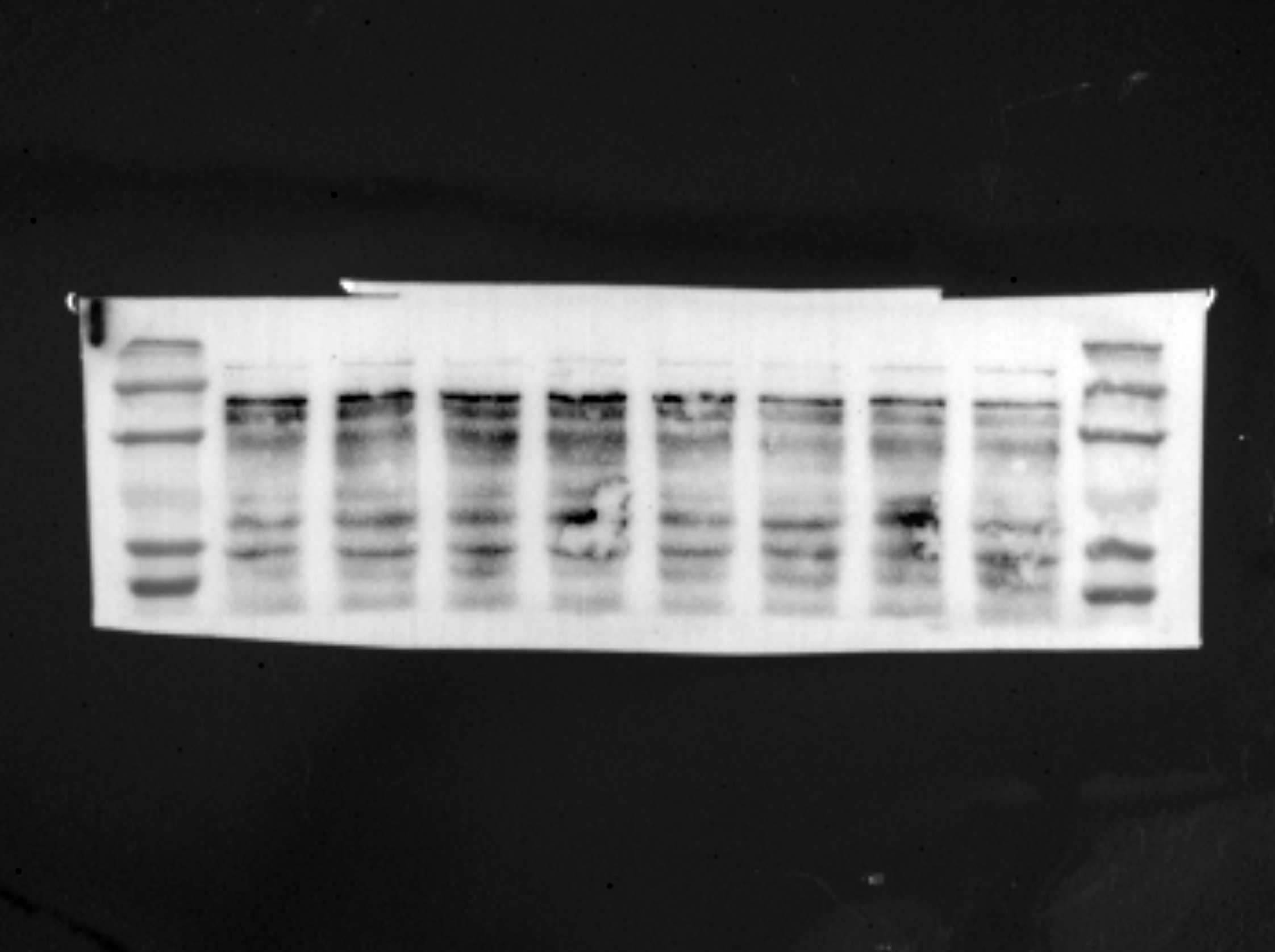

Supplement: S3 Image — (ZIP) [file pone.0325363.s003.zip › Raw_WB_Images/DNTTIP2-1.tif]

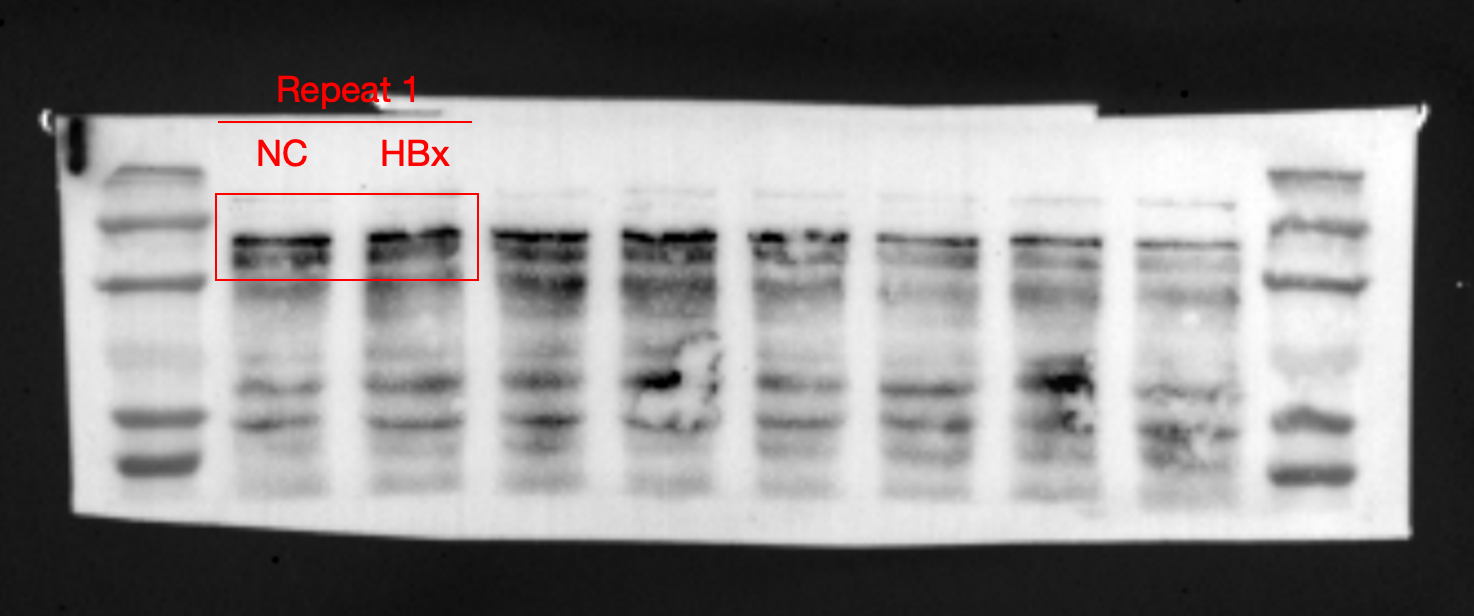

Supplement: S3 Image — (ZIP) [file pone.0325363.s003.zip › Raw_WB_Images/DNTTIP2-1 (with annotation).tif]

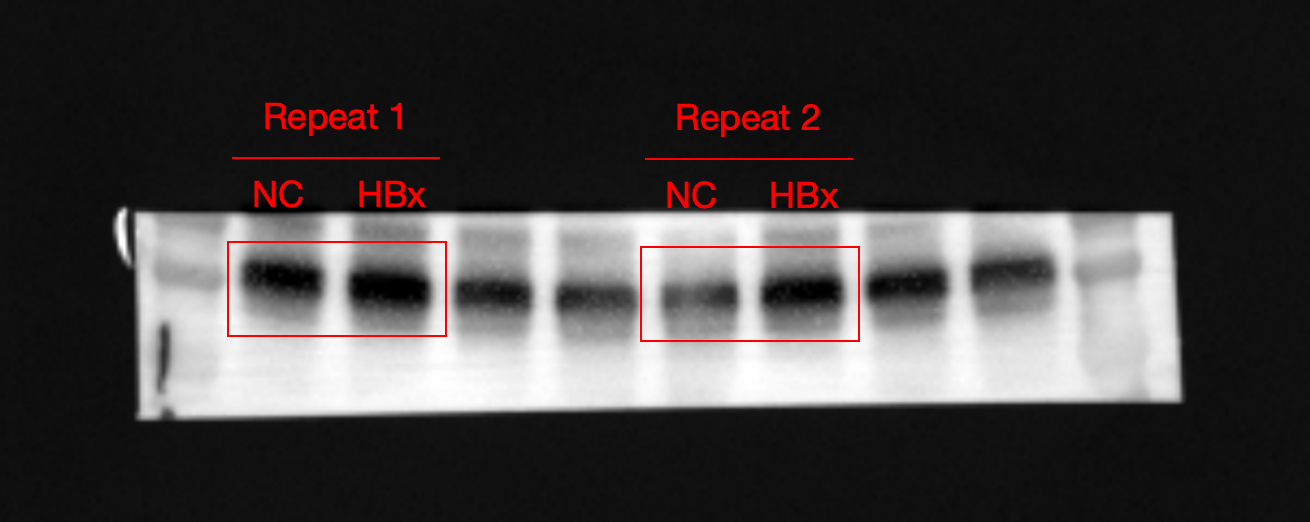

Supplement: S3 Image — (ZIP) [file pone.0325363.s003.zip › Raw_WB_Images/CD74-1 (with annotation).tif]

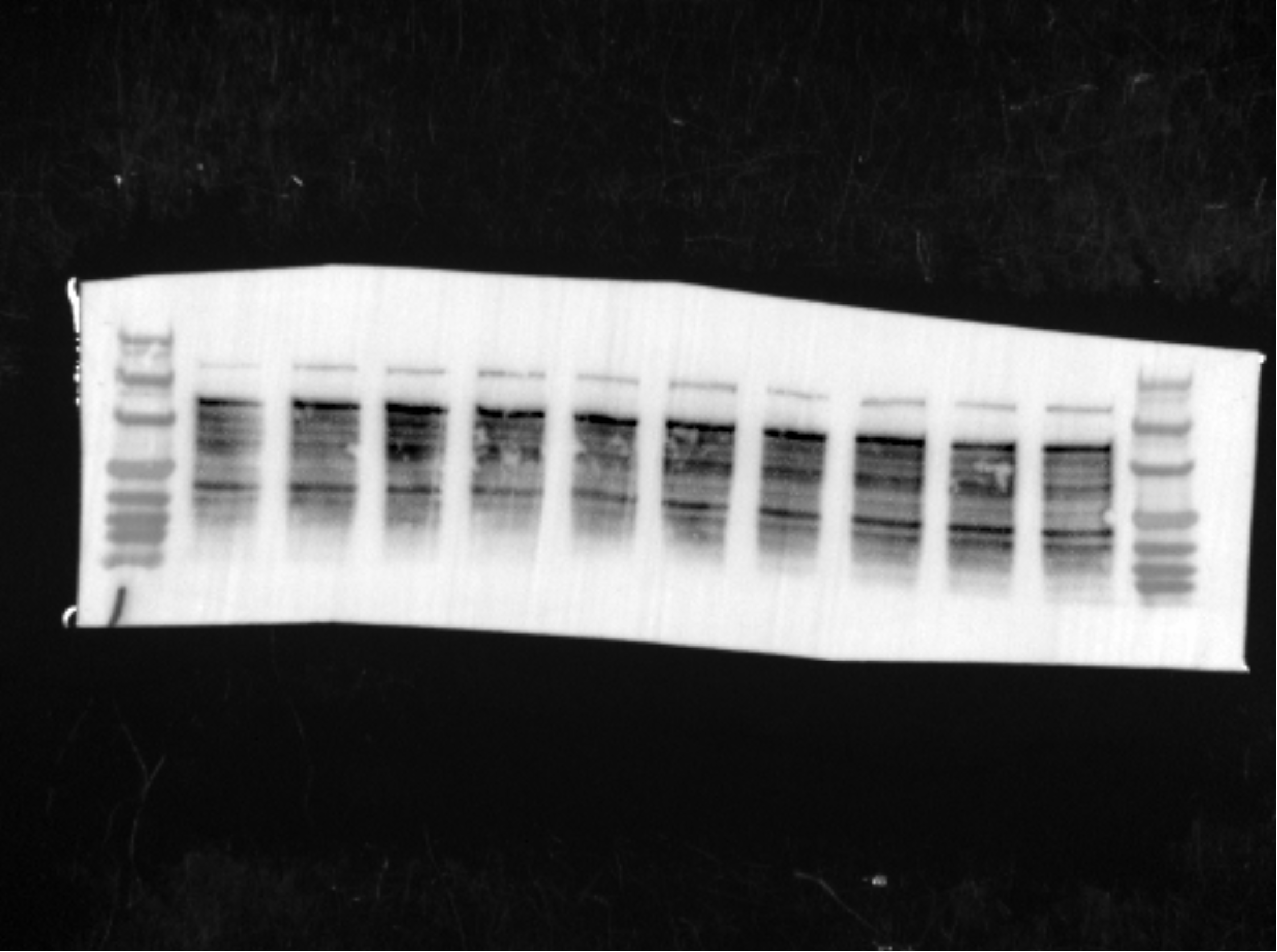

Supplement: S3 Image — (ZIP) [file pone.0325363.s003.zip › Raw_WB_Images/DNTTIP2-2.tif]

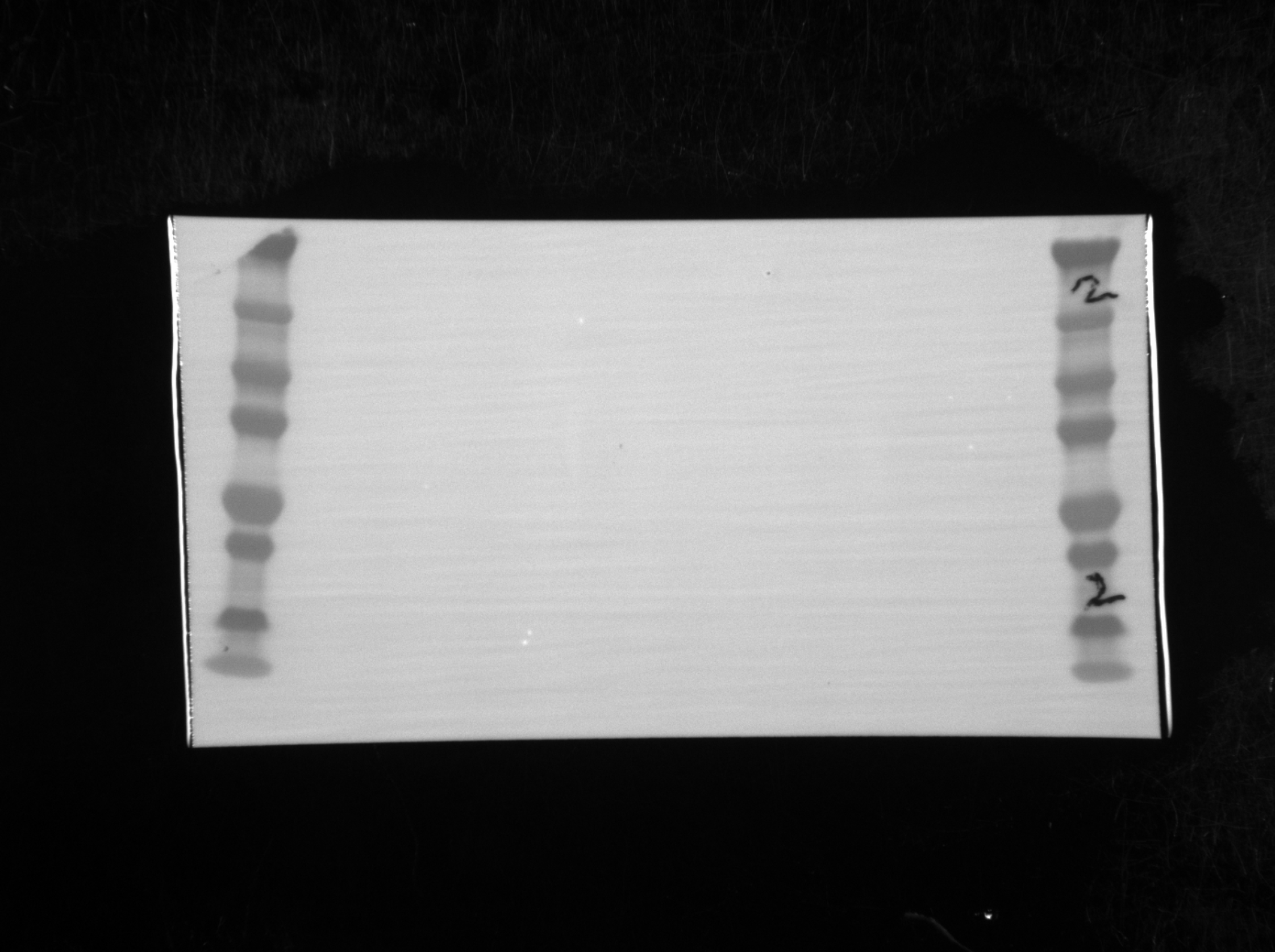

Supplement: S3 Image — (ZIP) [file pone.0325363.s003.zip › Raw_WB_Images/MIF-2 membrane (uncropped).tif]

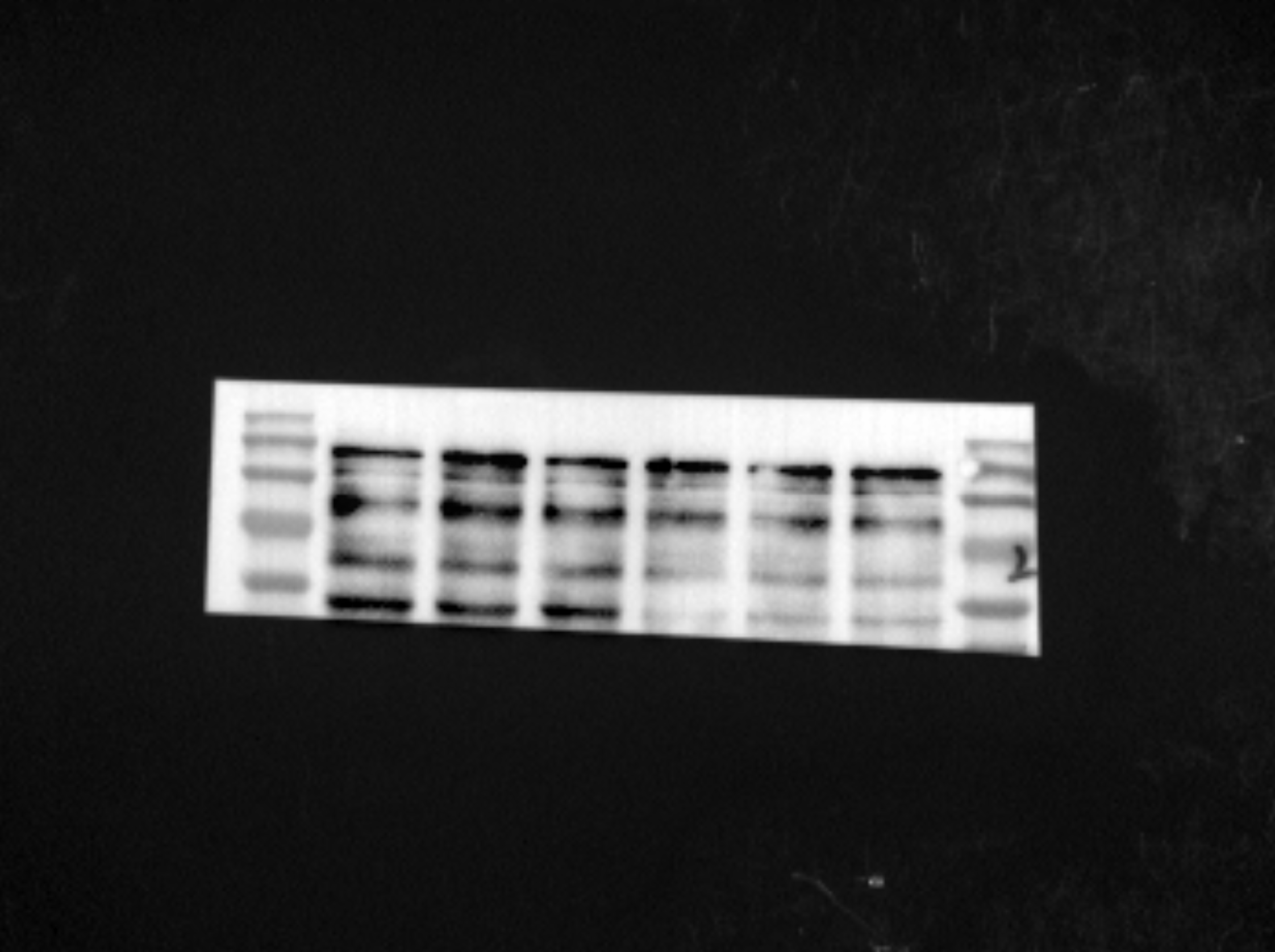

Supplement: S3 Image — (ZIP) [file pone.0325363.s003.zip › Raw_WB_Images/DNTTIP2-3.tif]

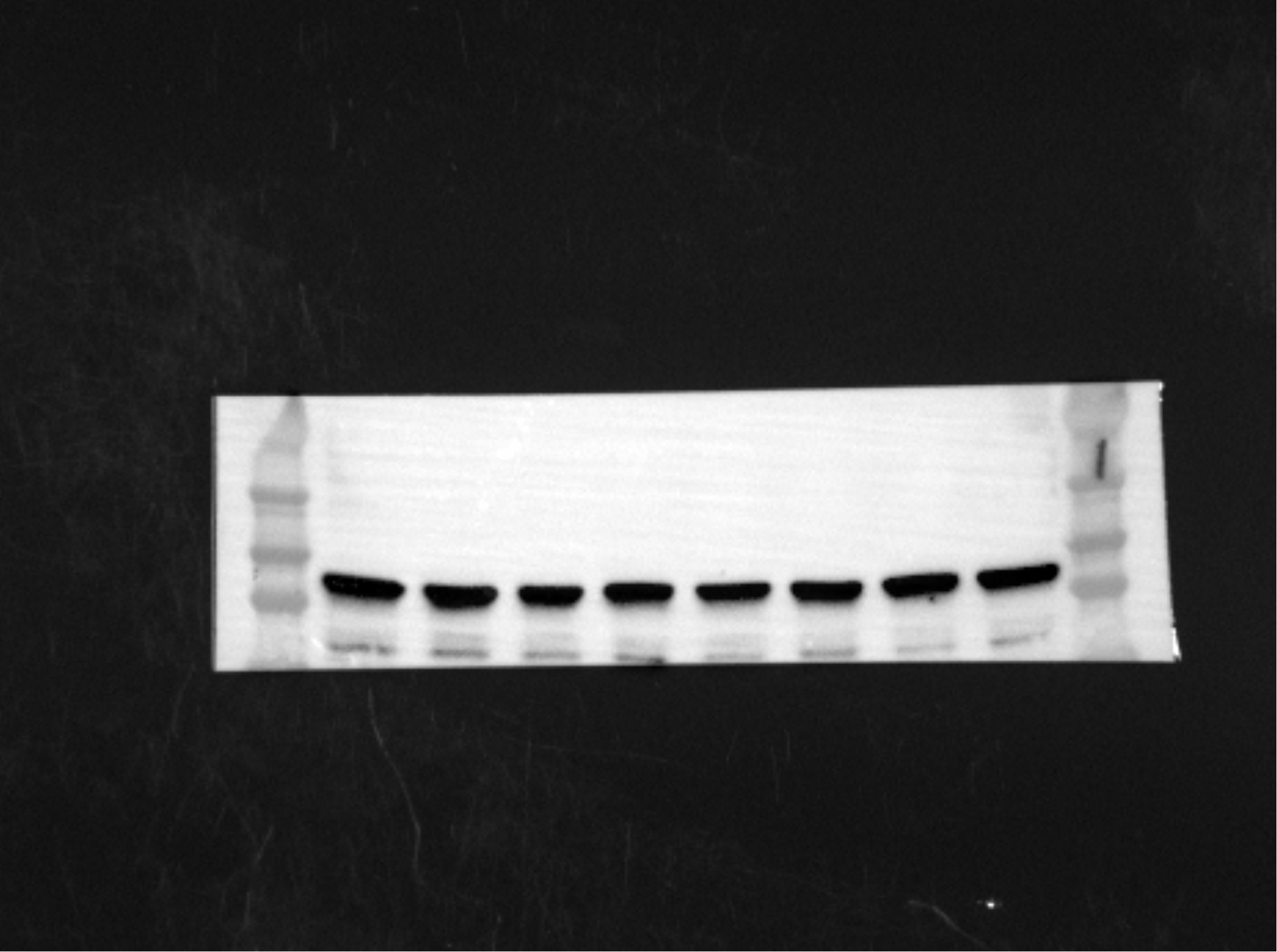

Supplement: S3 Image — (ZIP) [file pone.0325363.s003.zip › Raw_WB_Images/MIF-GAPDH-1 (loading control).tif]

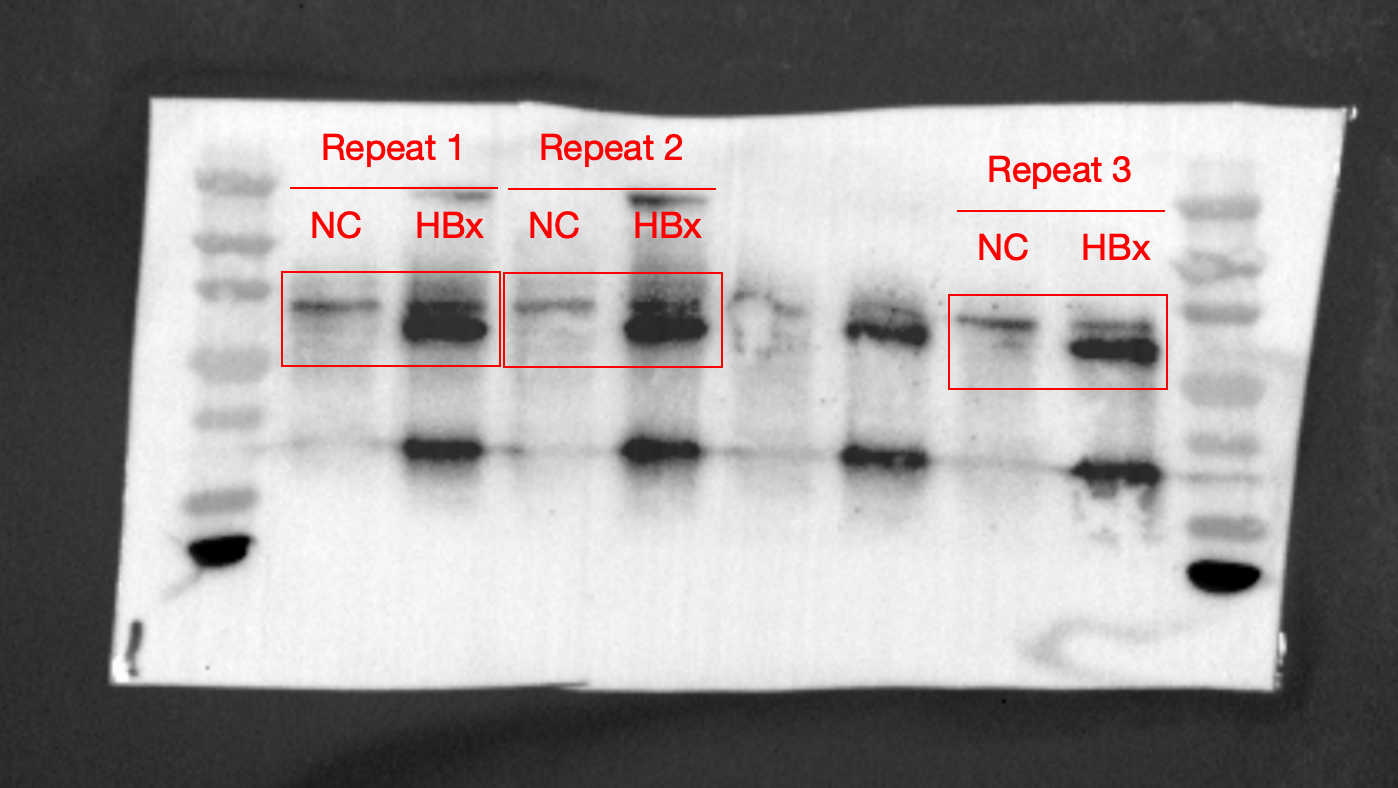

Supplement: S3 Image — (ZIP) [file pone.0325363.s003.zip › Raw_WB_Images/HBx (with annotation).tif]

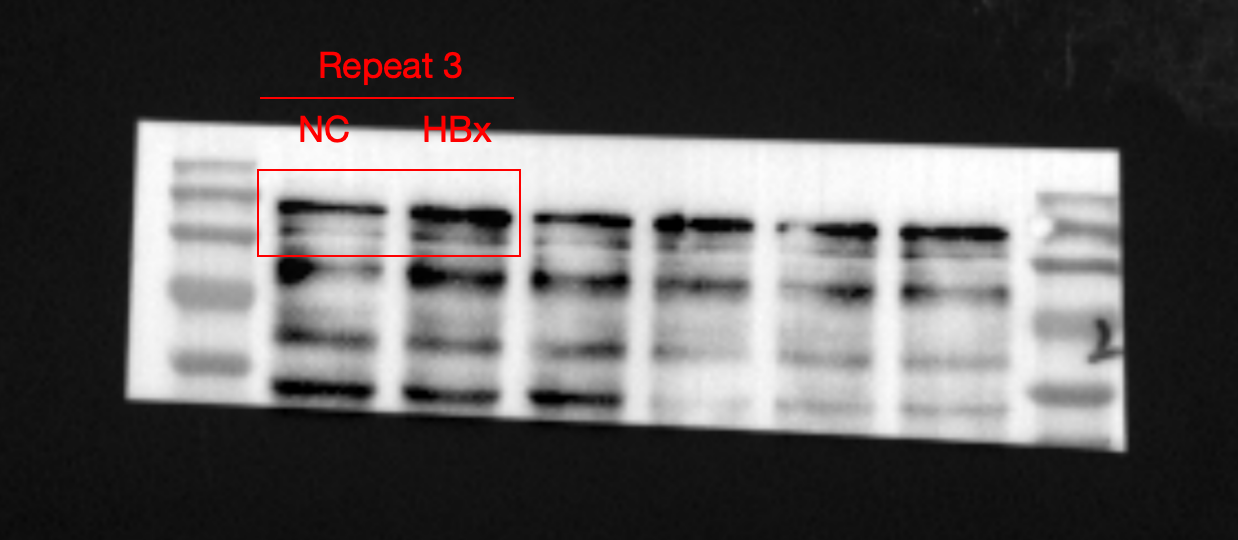

Supplement: S3 Image — (ZIP) [file pone.0325363.s003.zip › Raw_WB_Images/DNTTIP2-3 (with annotation).tif]

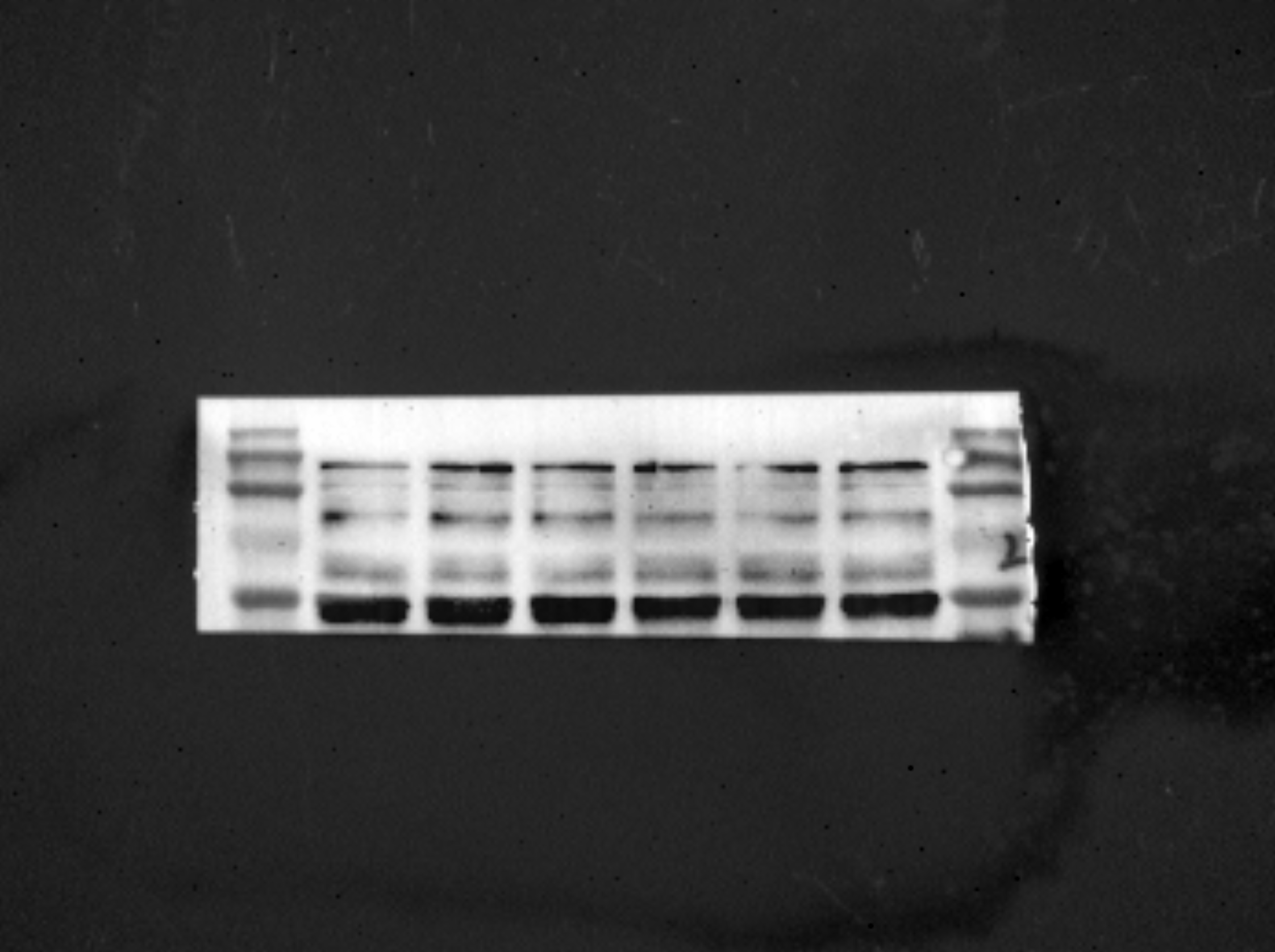

Supplement: S3 Image — (ZIP) [file pone.0325363.s003.zip › Raw_WB_Images/DNTTIP2-tublin-3 (loading control).tif]

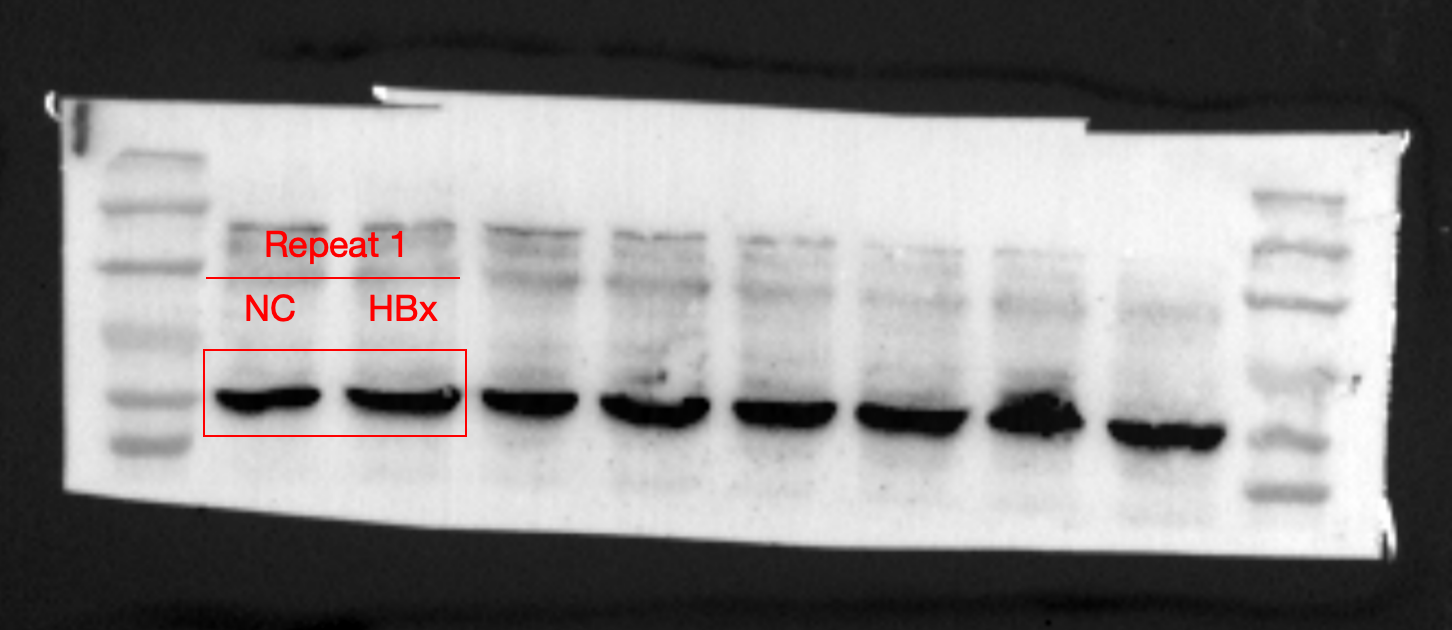

Supplement: S3 Image — (ZIP) [file pone.0325363.s003.zip › Raw_WB_Images/DNTTIP2-tublin-1 (loading control) (with annotation).tif]

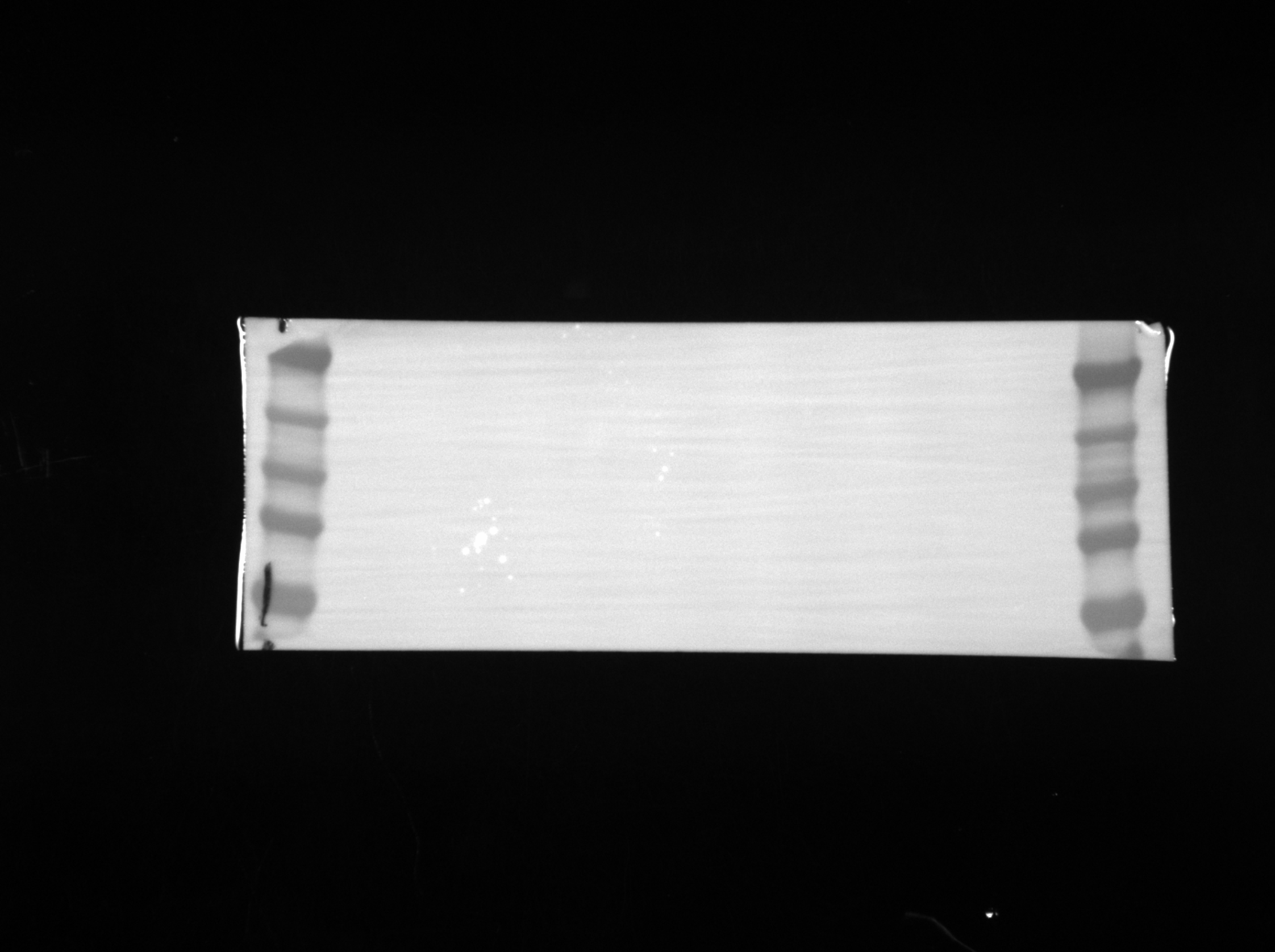

Supplement: S3 Image — (ZIP) [file pone.0325363.s003.zip › Raw_WB_Images/CD74-1 membrane (uncropped).tif]

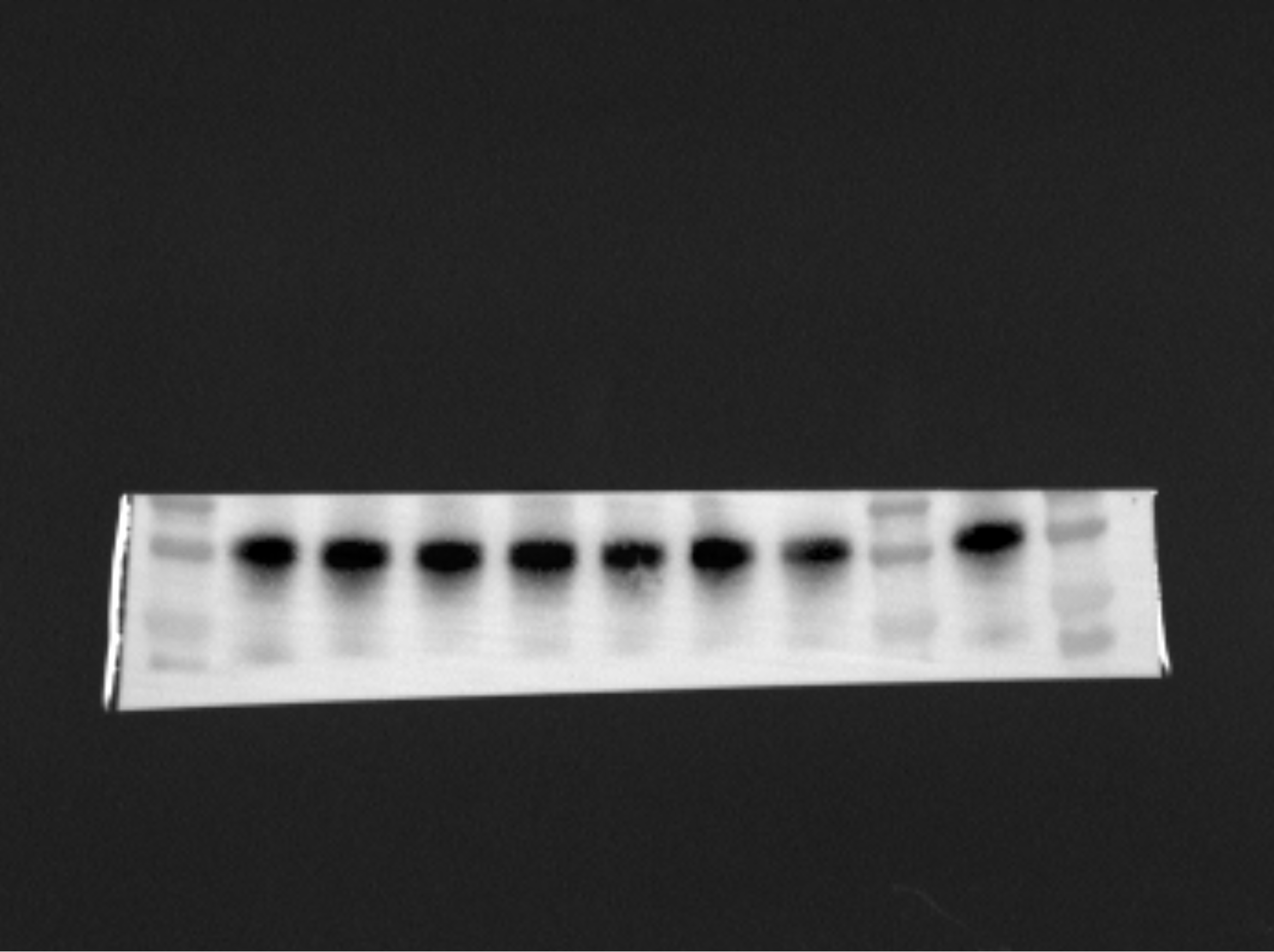

Supplement: S3 Image — (ZIP) [file pone.0325363.s003.zip › Raw_WB_Images/CD74-2.tif]

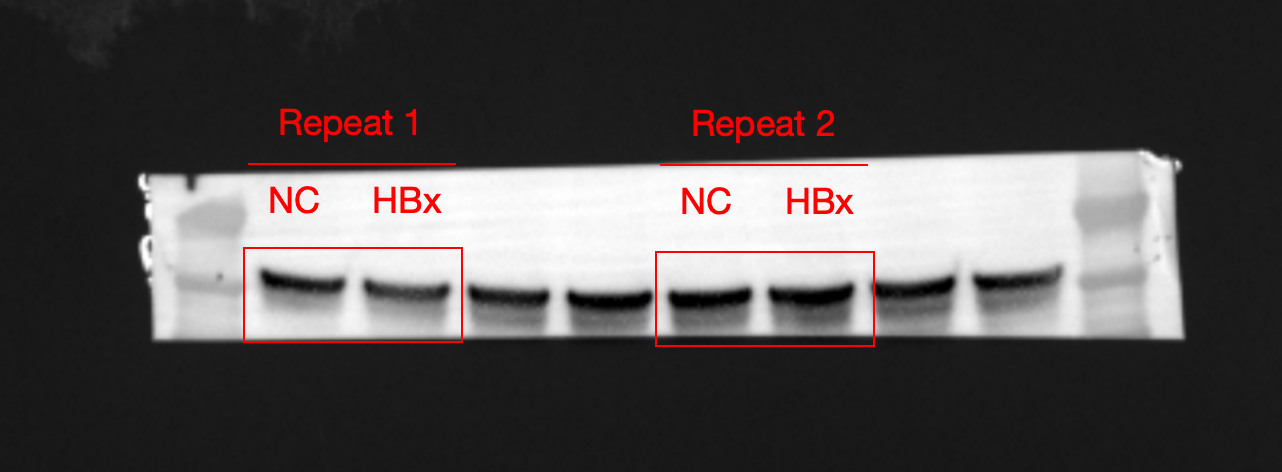

Supplement: S3 Image — (ZIP) [file pone.0325363.s003.zip › Raw_WB_Images/CD74-tublin-1 (loading control) (with annotation).tif]

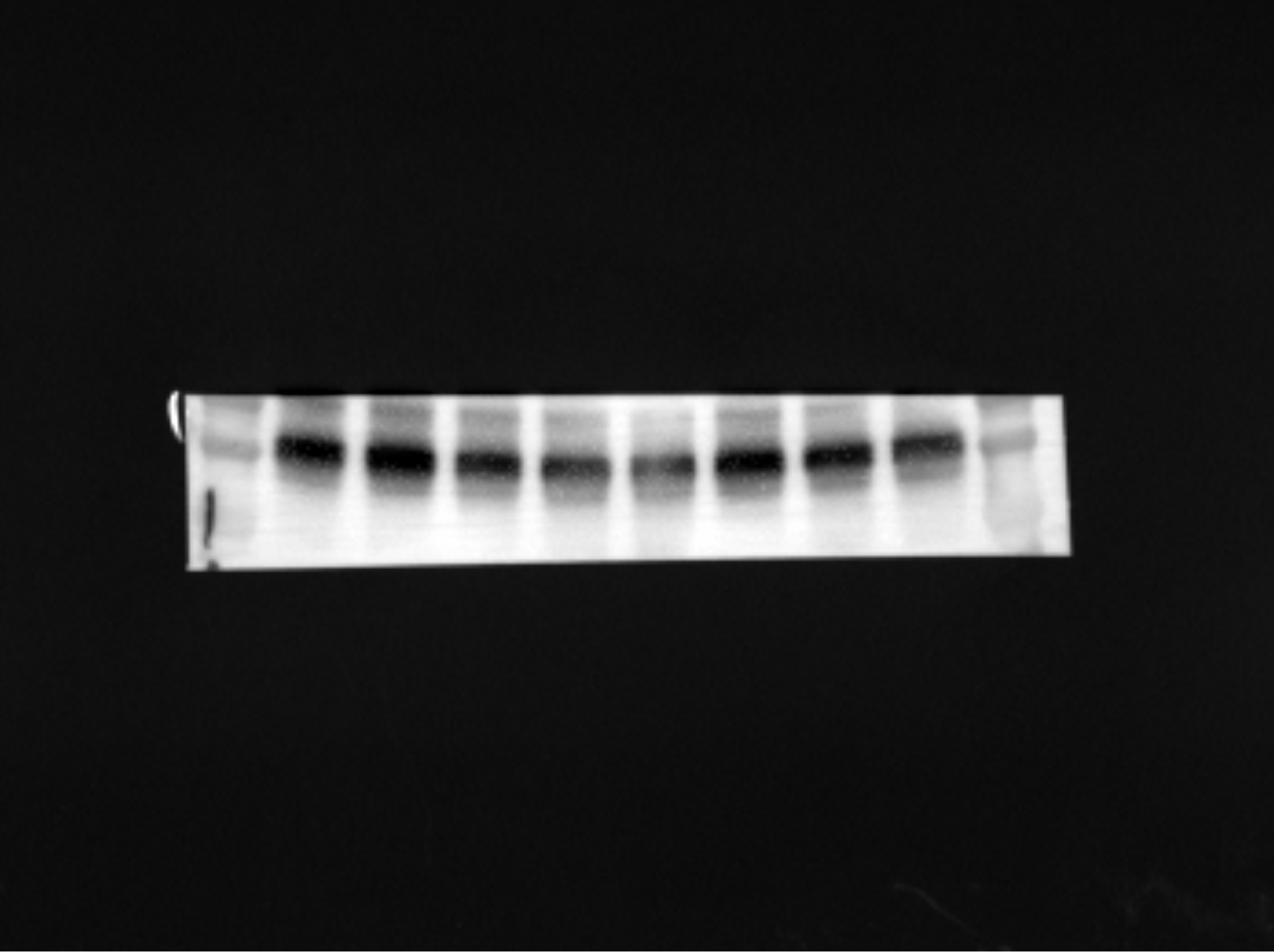

Supplement: S3 Image — (ZIP) [file pone.0325363.s003.zip › Raw_WB_Images/CD74-1.tif]

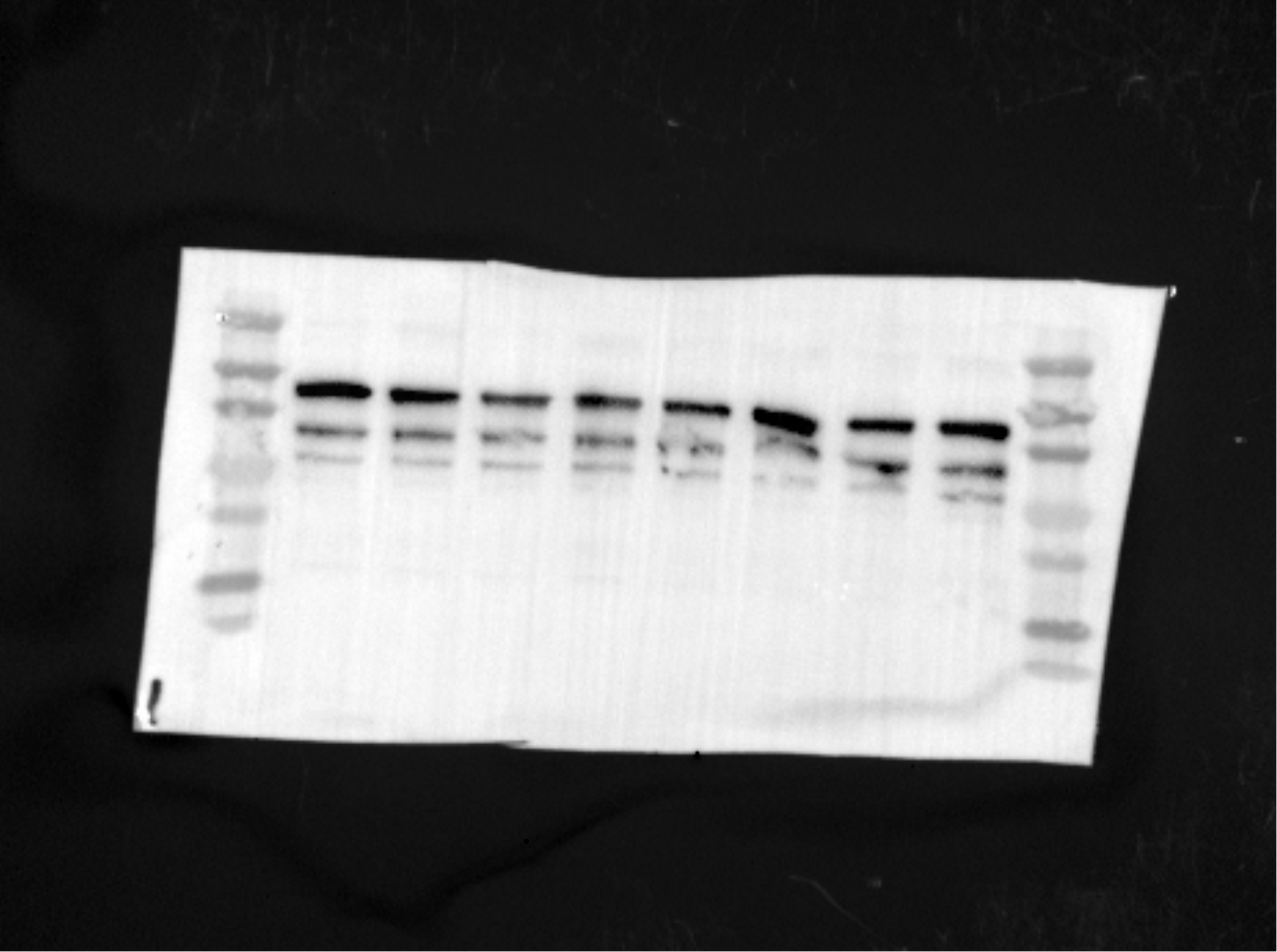

Supplement: S3 Image — (ZIP) [file pone.0325363.s003.zip › Raw_WB_Images/HBx-GAPDH (loading control).tif]

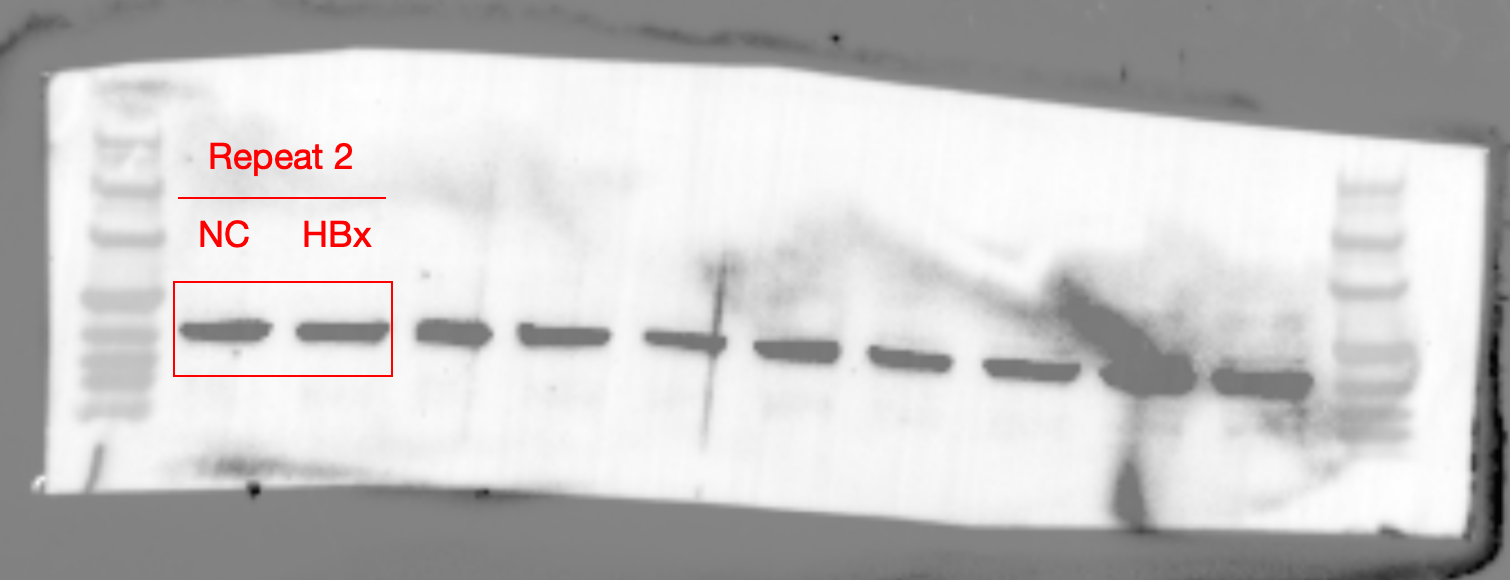

Supplement: S3 Image — (ZIP) [file pone.0325363.s003.zip › Raw_WB_Images/DNTTIP2-tublin-2 (loading control) (with annotation).tif]

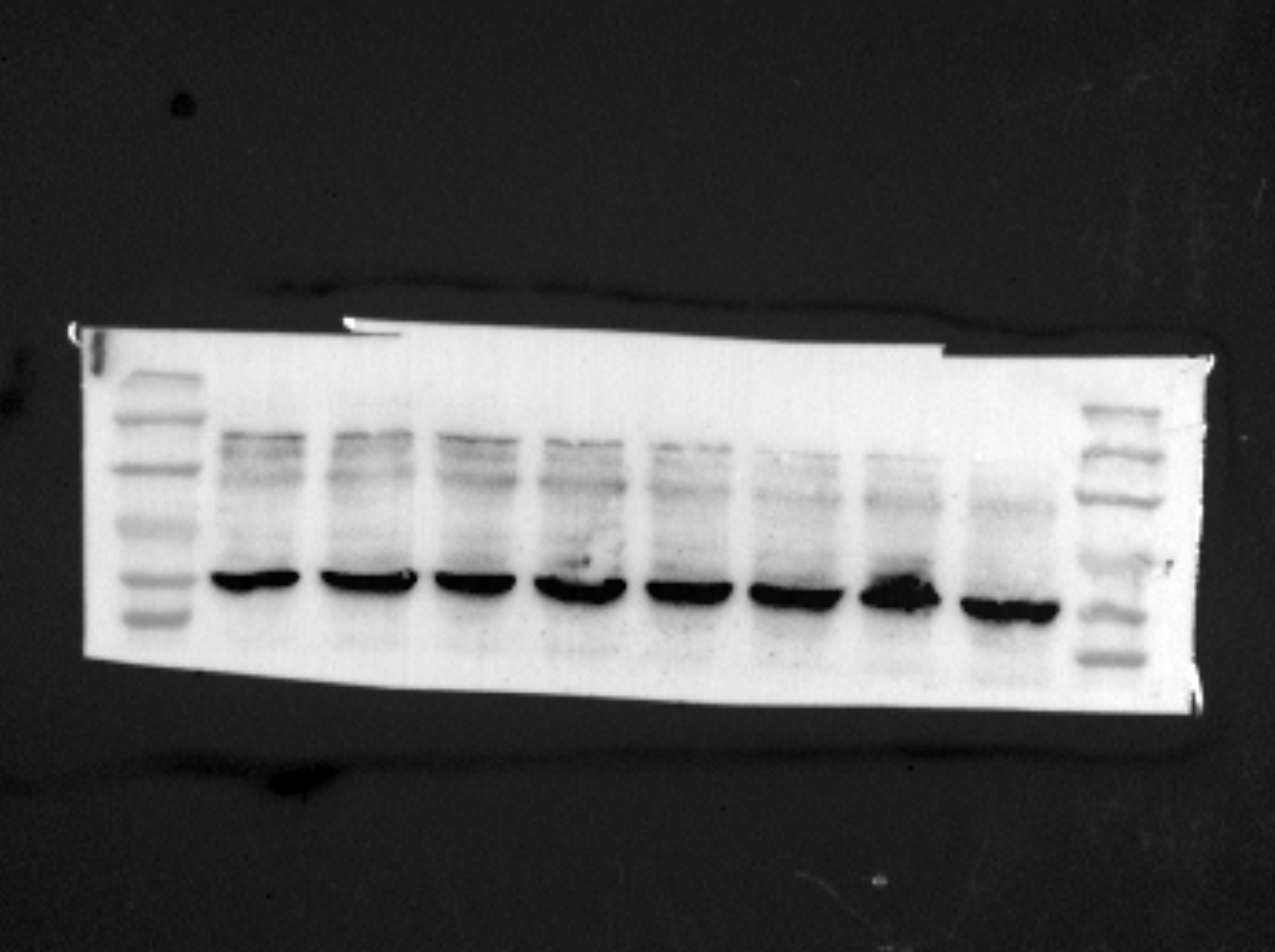

Supplement: S3 Image — (ZIP) [file pone.0325363.s003.zip › Raw_WB_Images/DNTTIP2-tublin-1 (loading control).tif]

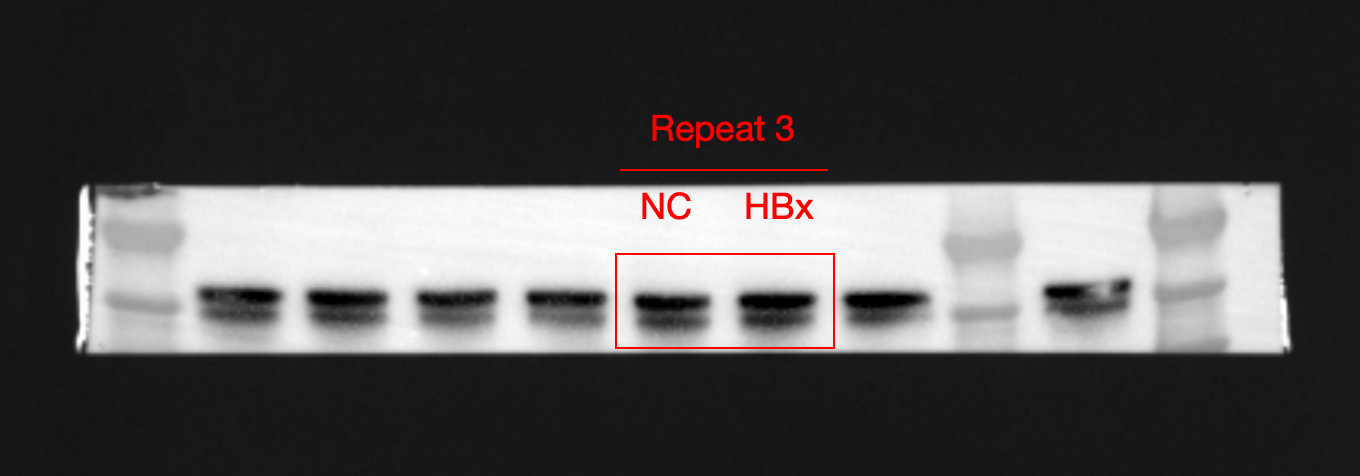

Supplement: S3 Image — (ZIP) [file pone.0325363.s003.zip › Raw_WB_Images/CD74-tublin-2 (loading control) (with annotation).tif]

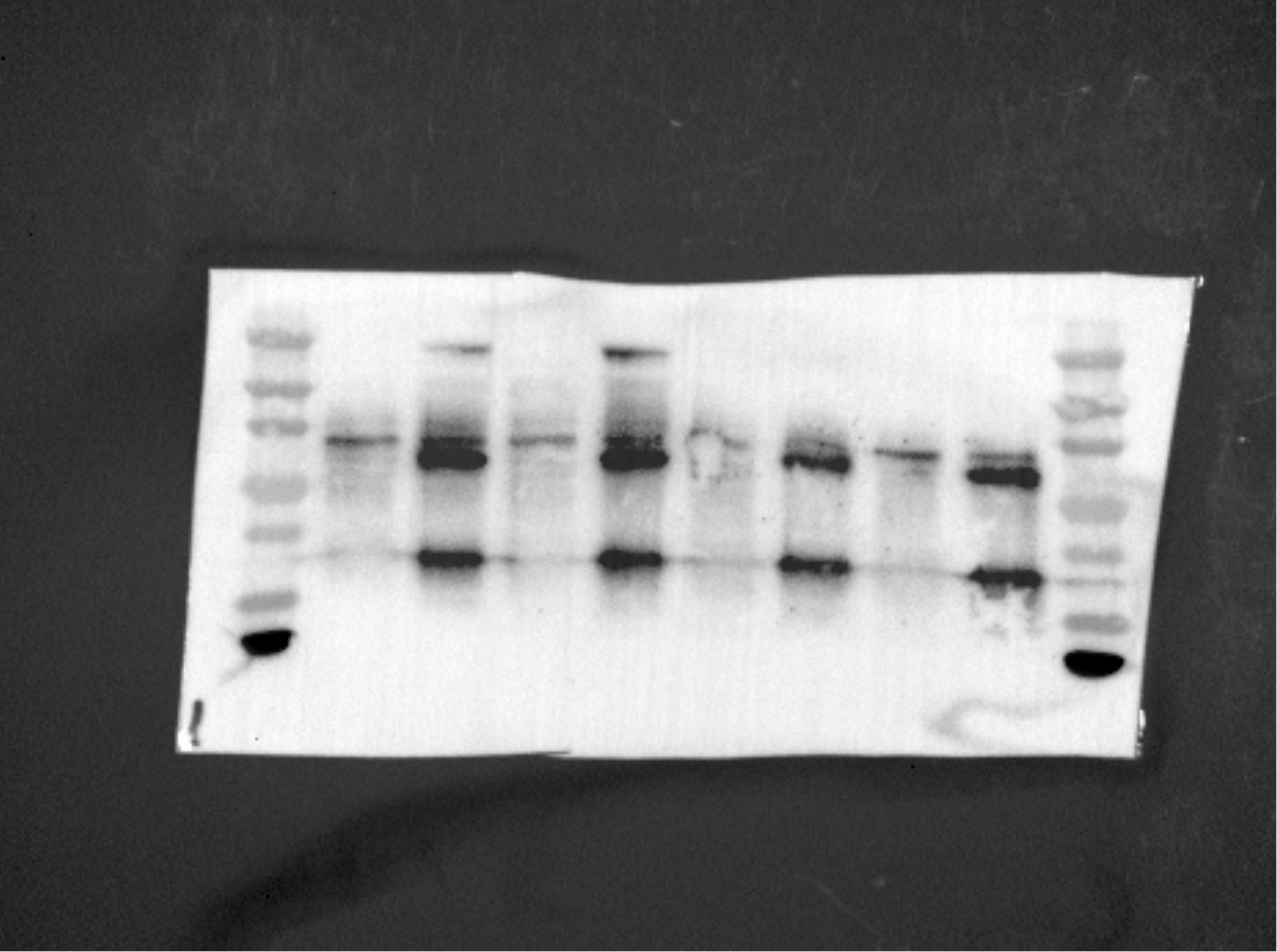

Supplement: S3 Image — (ZIP) [file pone.0325363.s003.zip › Raw_WB_Images/HBx.tif]

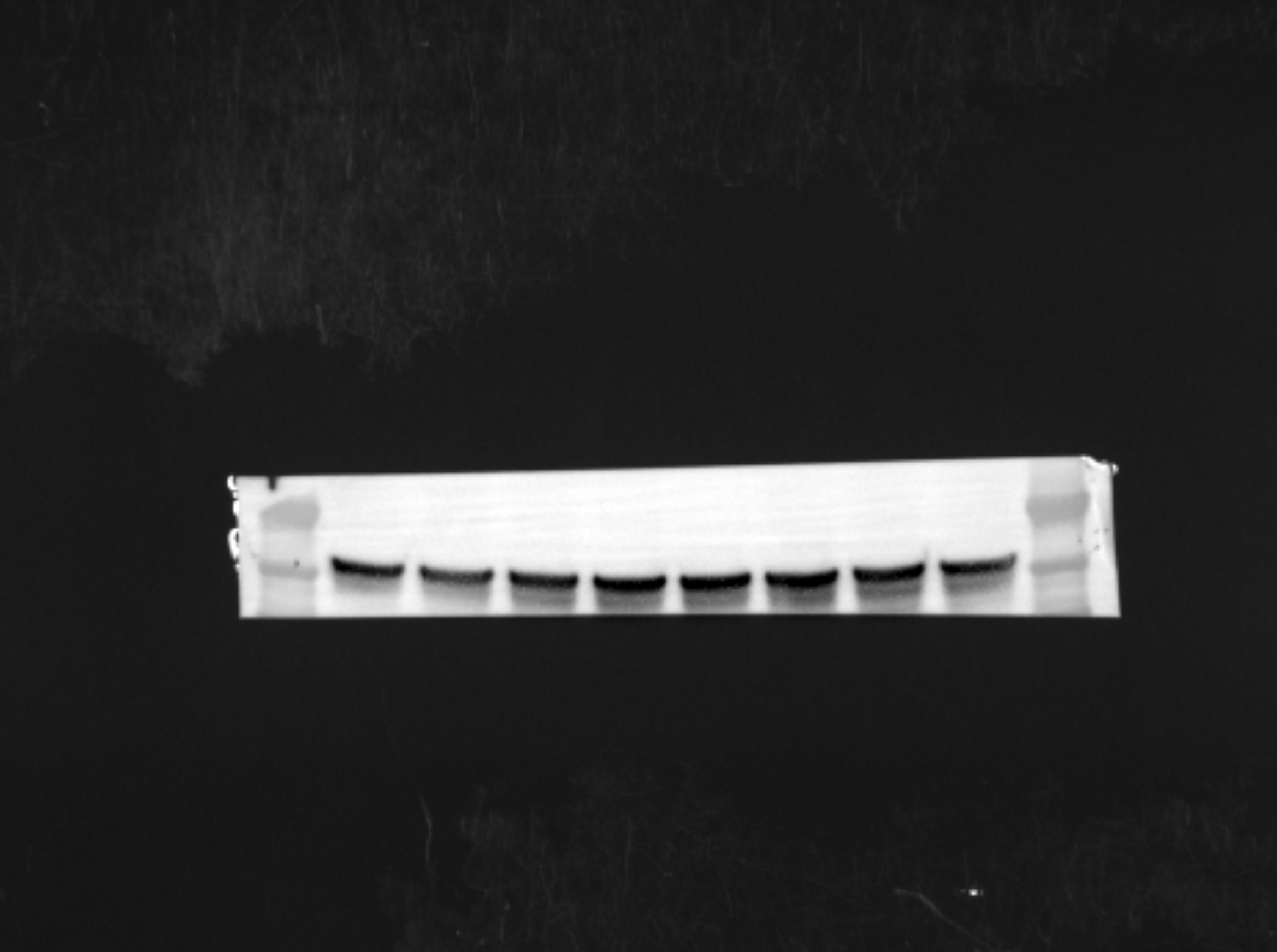

Supplement: S3 Image — (ZIP) [file pone.0325363.s003.zip › Raw_WB_Images/CD74-tublin-1 (loading control).tif]

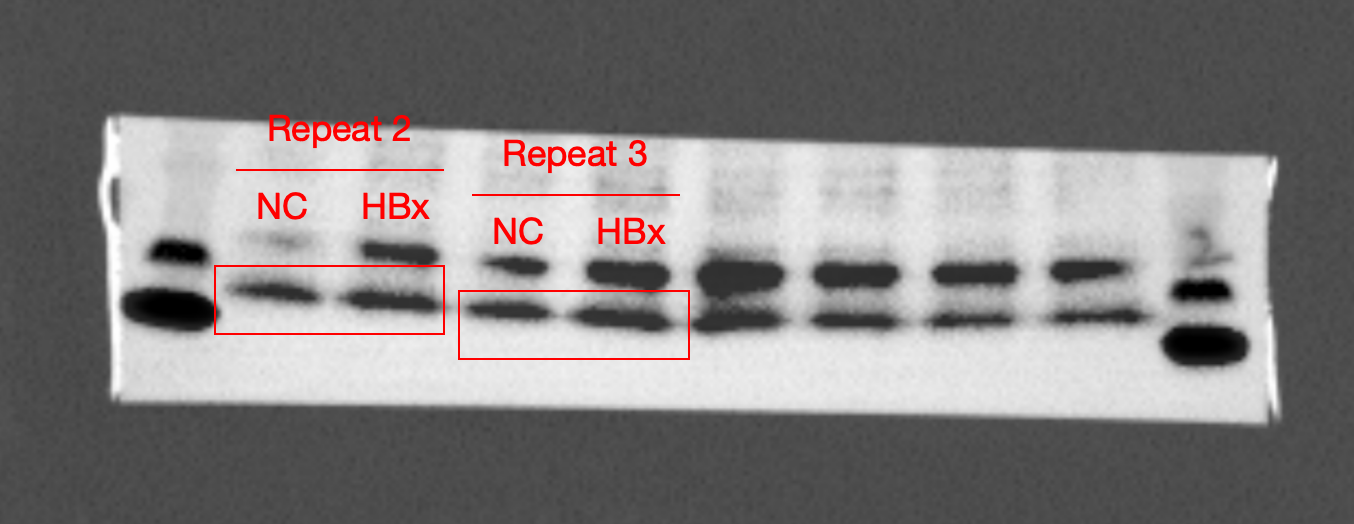

Supplement: S3 Image — (ZIP) [file pone.0325363.s003.zip › Raw_WB_Images/MIF-2 (with annotation).tif]

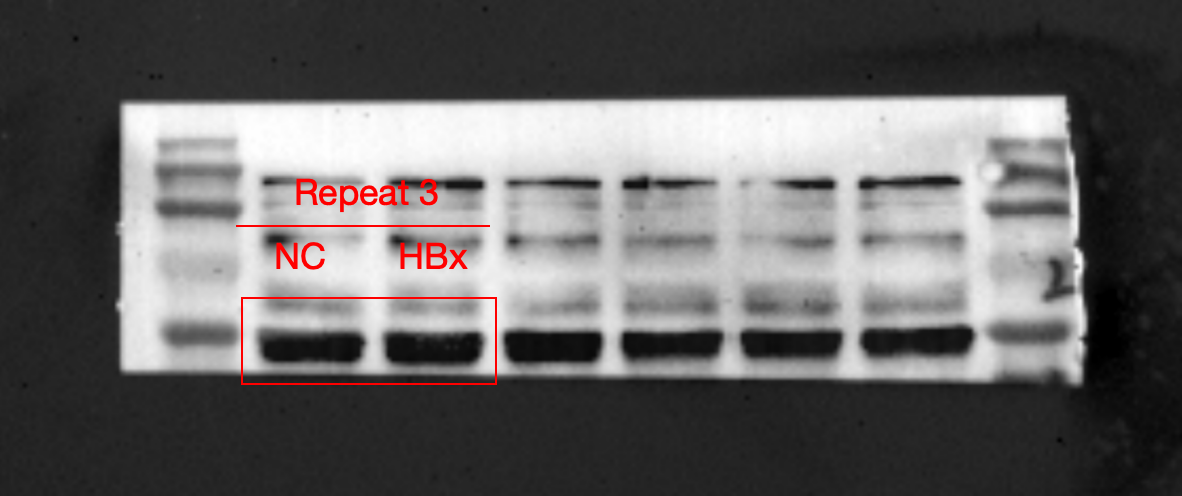

Supplement: S3 Image — (ZIP) [file pone.0325363.s003.zip › Raw_WB_Images/DNTTIP2-tublin-3 (loading control) (with annotation).tif]

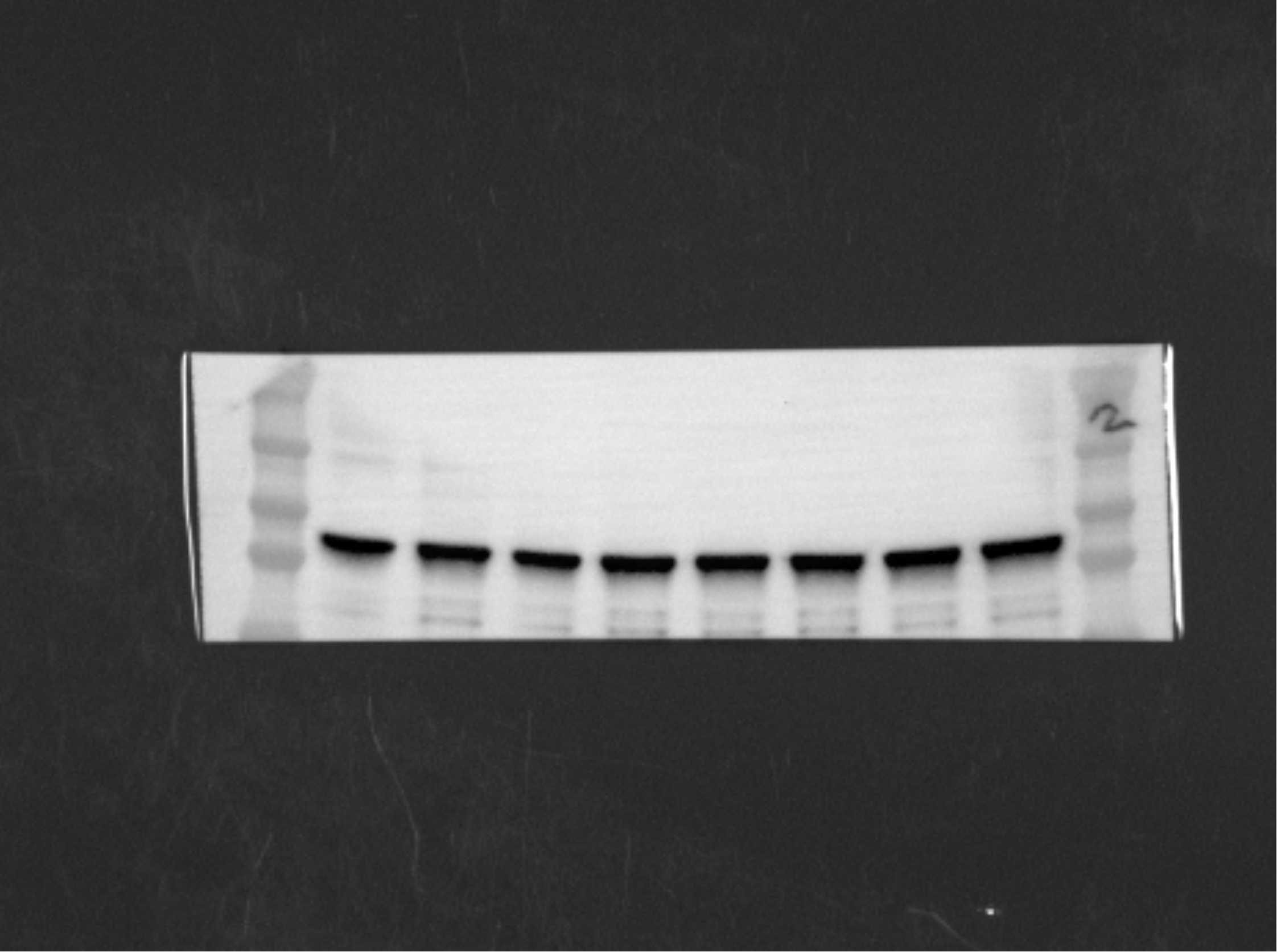

Supplement: S3 Image — (ZIP) [file pone.0325363.s003.zip › Raw_WB_Images/MIF-GAPDH-2 (loading control).tif]

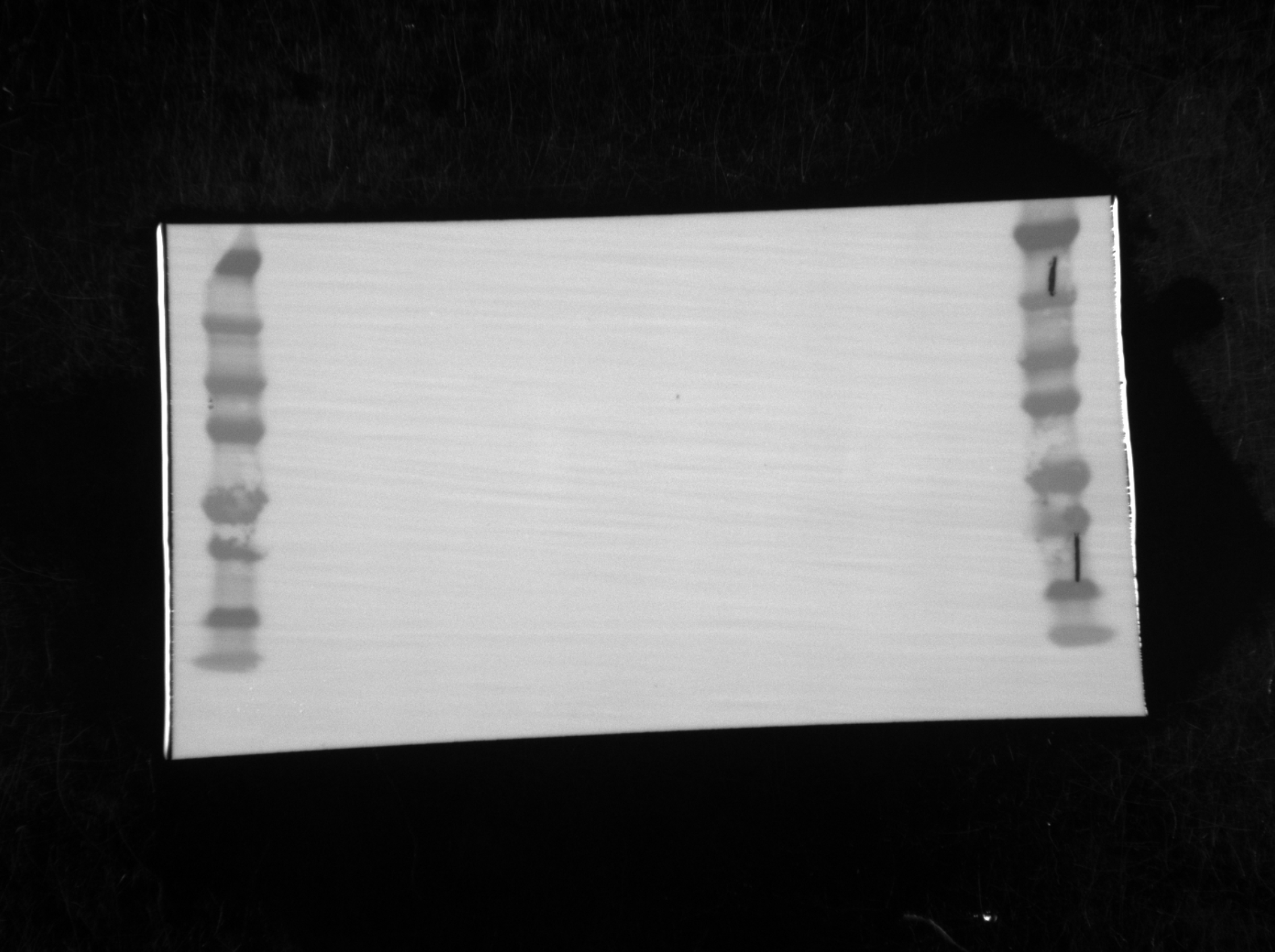

Supplement: S3 Image — (ZIP) [file pone.0325363.s003.zip › Raw_WB_Images/MIF-1 membrane (uncropped).tif]

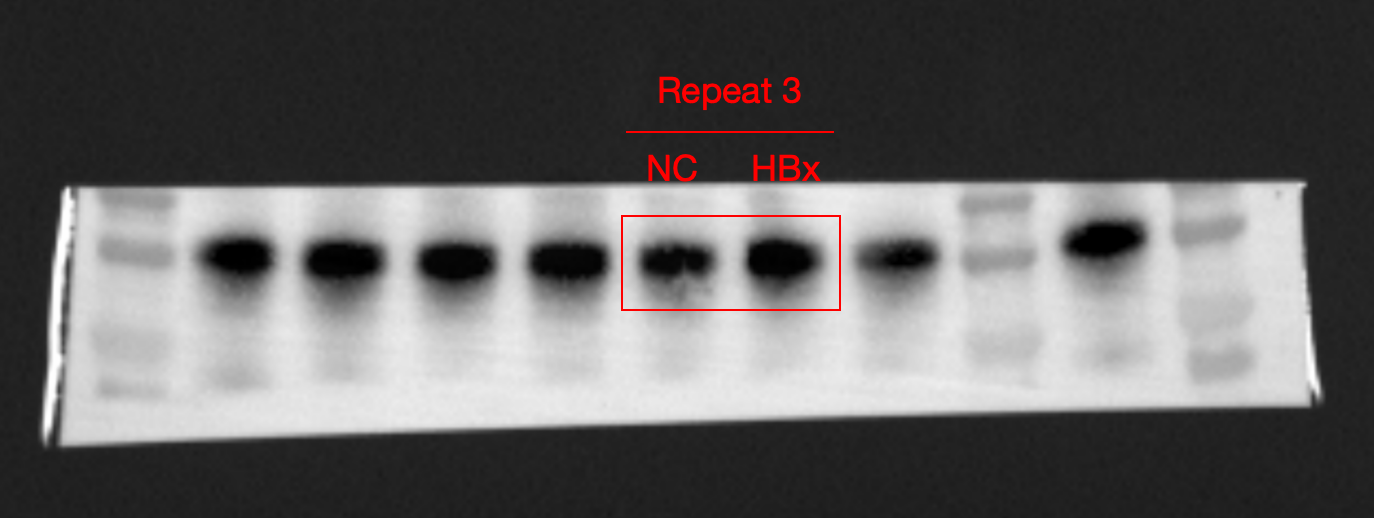

Supplement: S3 Image — (ZIP) [file pone.0325363.s003.zip › Raw_WB_Images/CD74-2 (with annotation).tif]

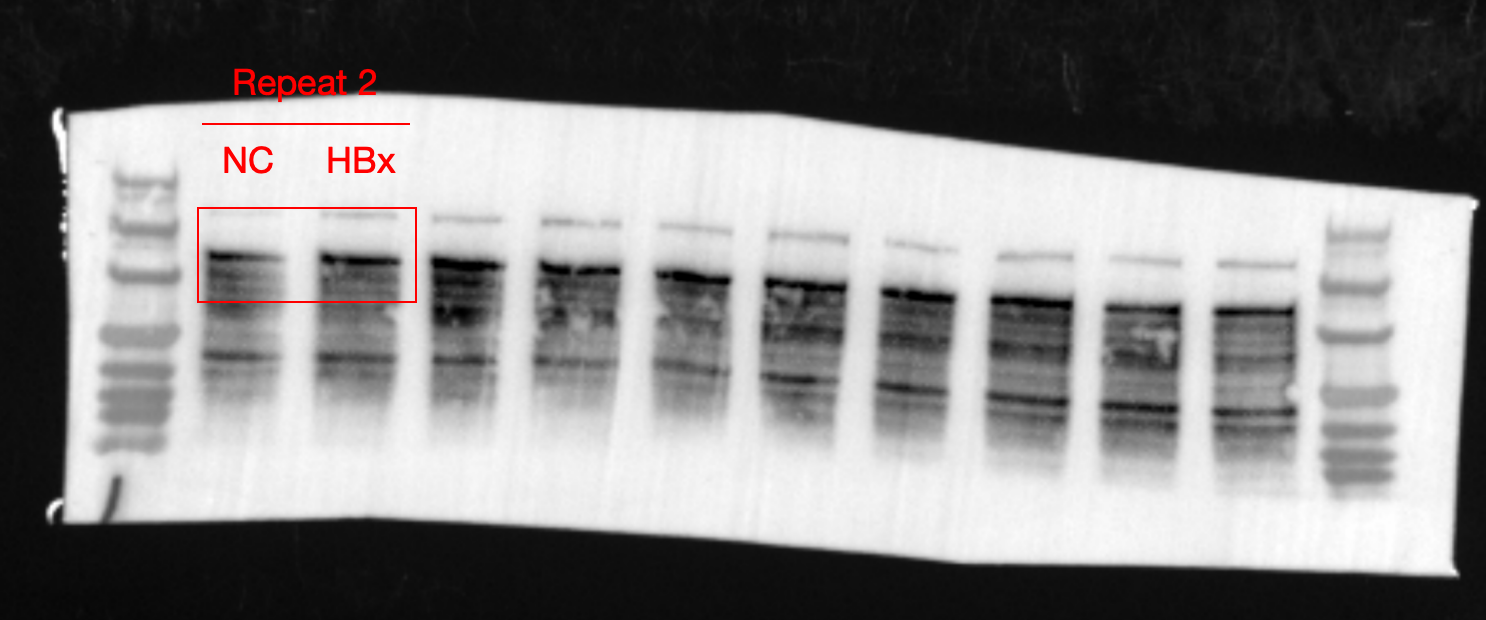

Supplement: S3 Image — (ZIP) [file pone.0325363.s003.zip › Raw_WB_Images/DNTTIP2-2 (with annotation).tif]

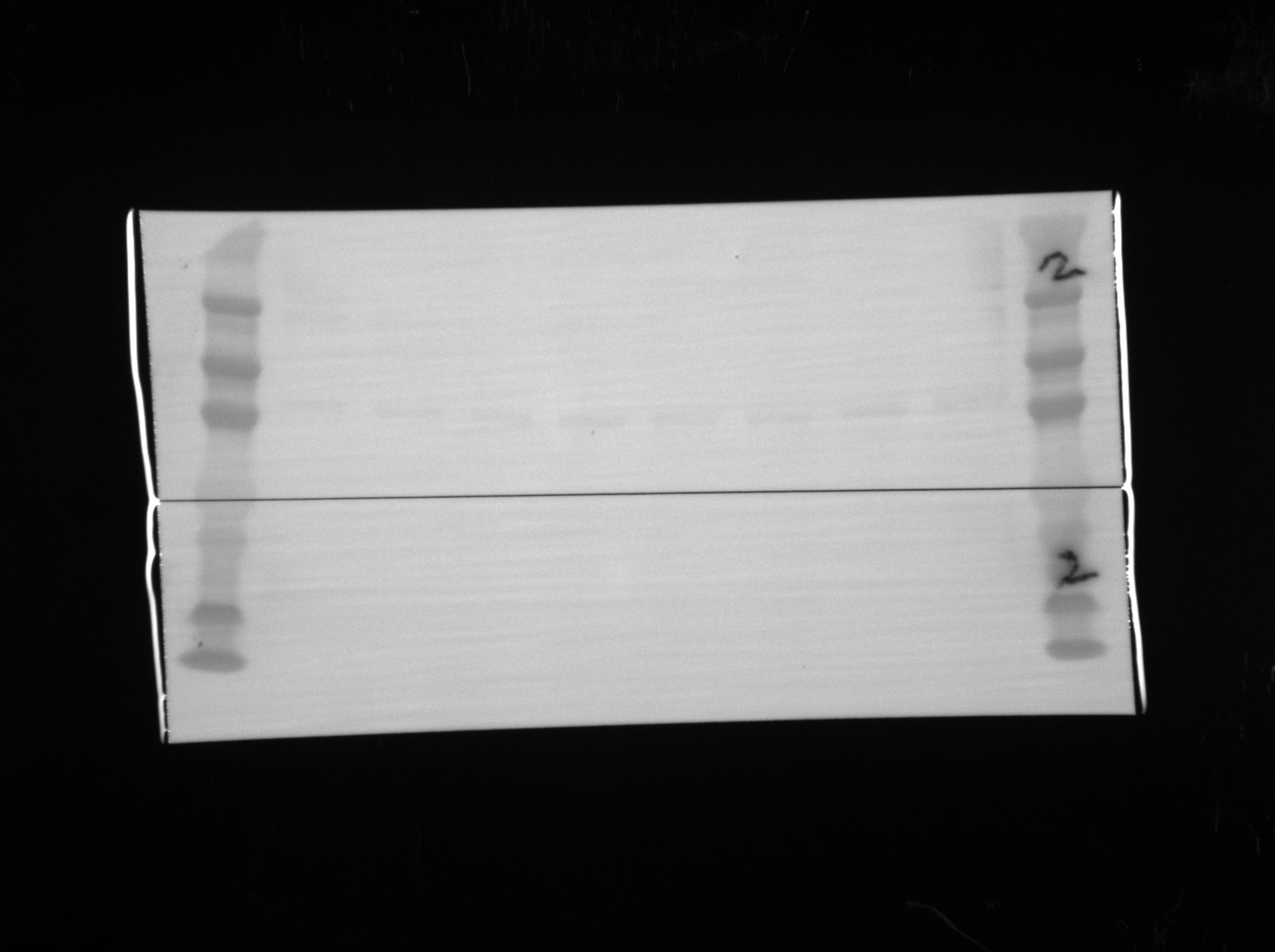

Supplement: S3 Image — (ZIP) [file pone.0325363.s003.zip › Raw_WB_Images/MIF-2 membrane (spliced).tif]

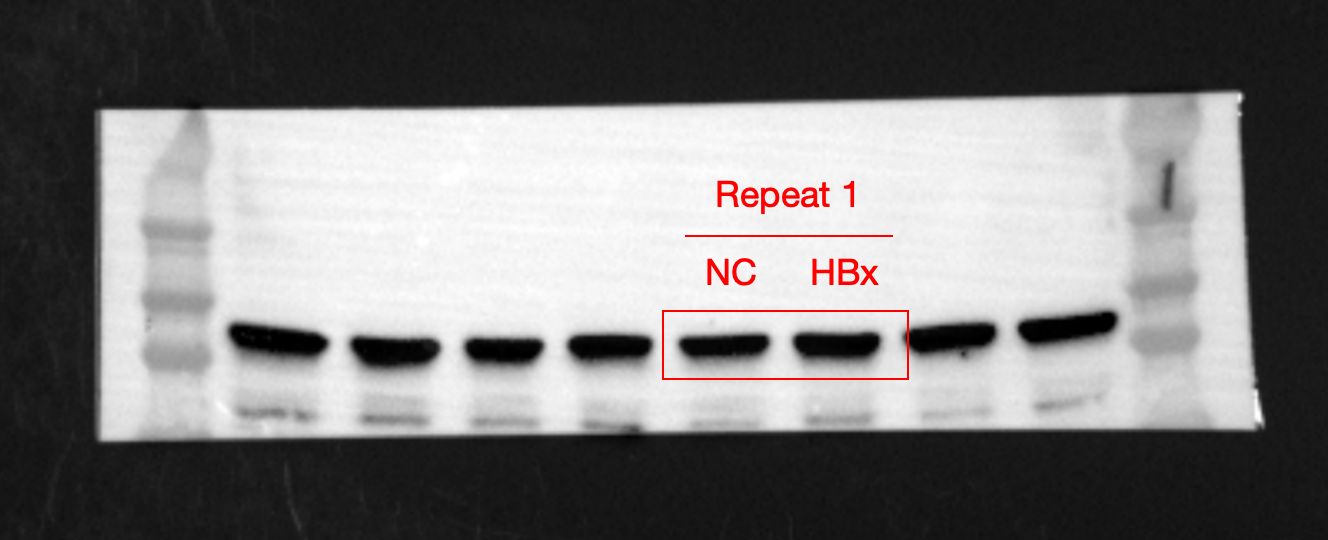

Supplement: S3 Image — (ZIP) [file pone.0325363.s003.zip › Raw_WB_Images/MIF-GAPDH-1 (loading control) (with annotation).tif]

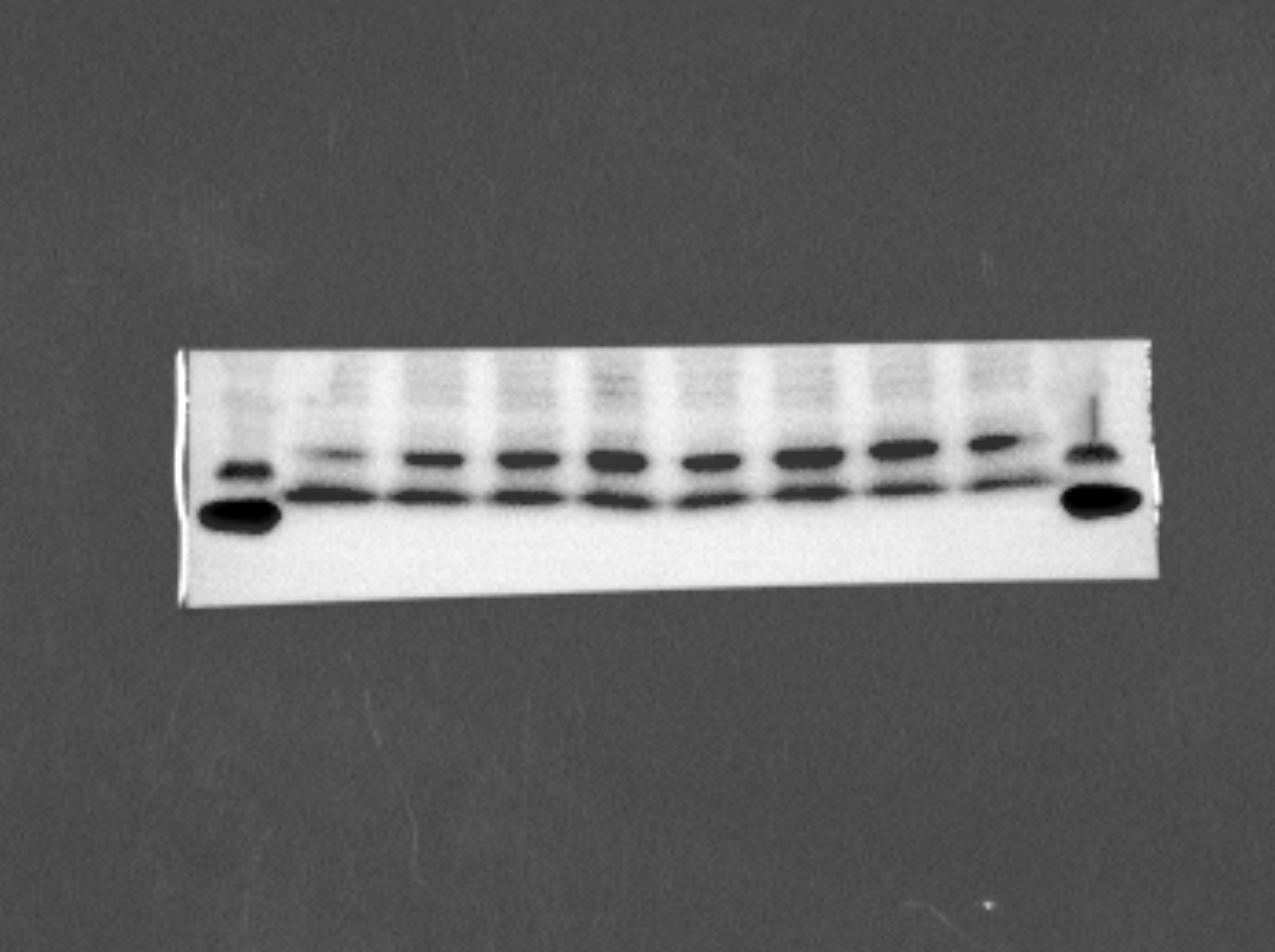

Supplement: S3 Image — (ZIP) [file pone.0325363.s003.zip › Raw_WB_Images/MIF-1.tif]

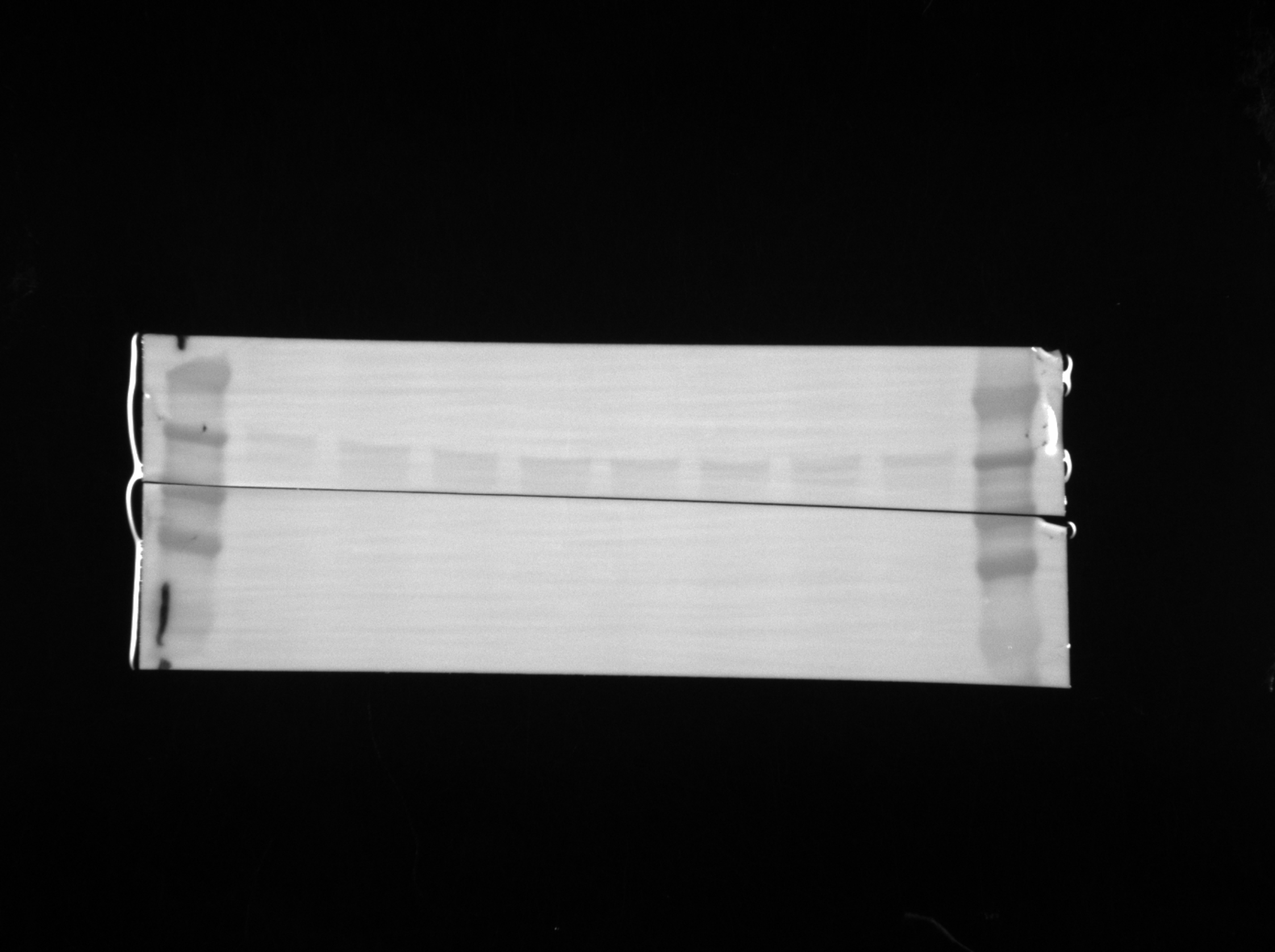

Supplement: S3 Image — (ZIP) [file pone.0325363.s003.zip › Raw_WB_Images/CD74-1 membrane (spliced).tif]

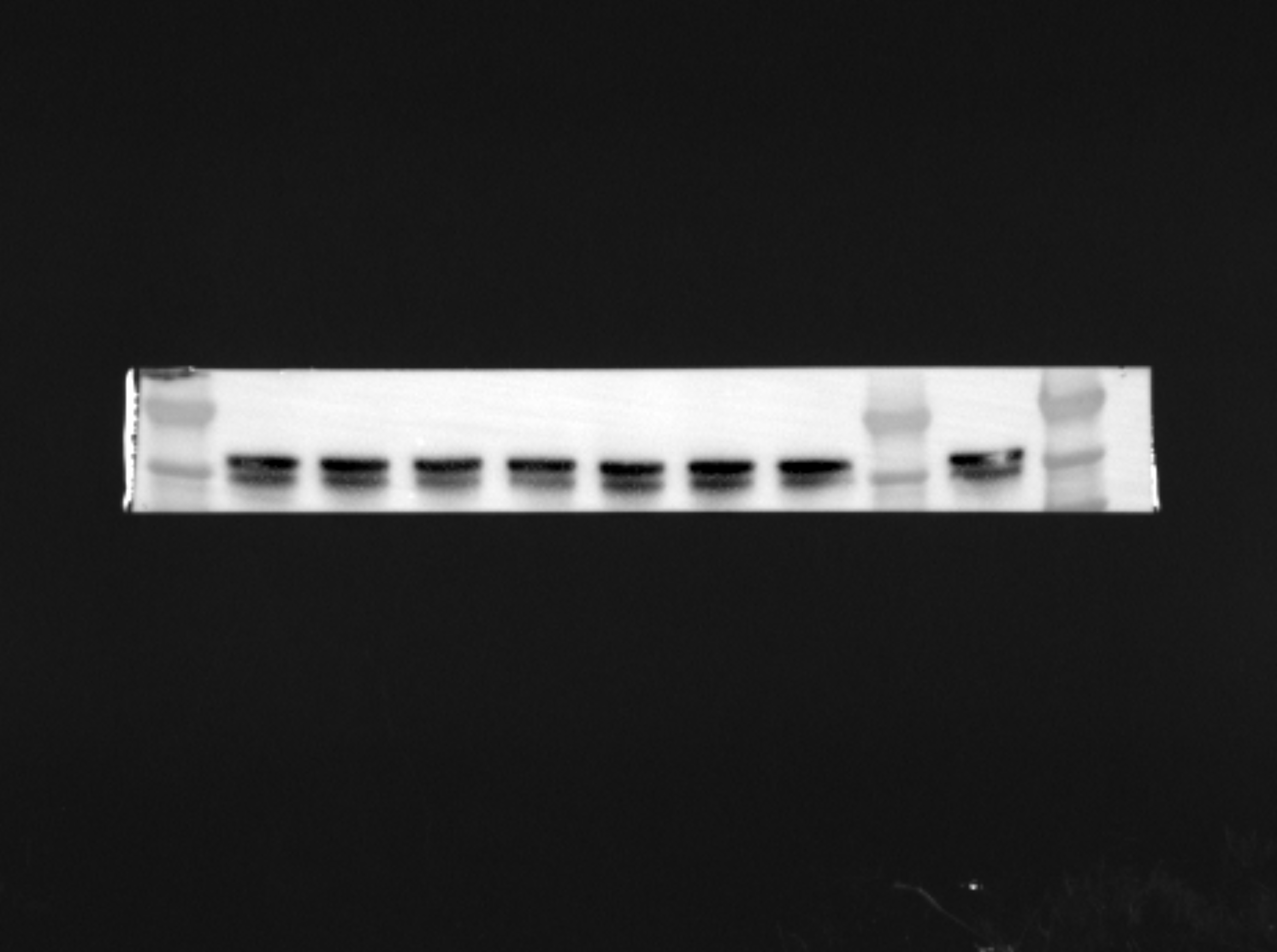

Supplement: S3 Image — (ZIP) [file pone.0325363.s003.zip › Raw_WB_Images/CD74-tublin-2 (loading control).tif]

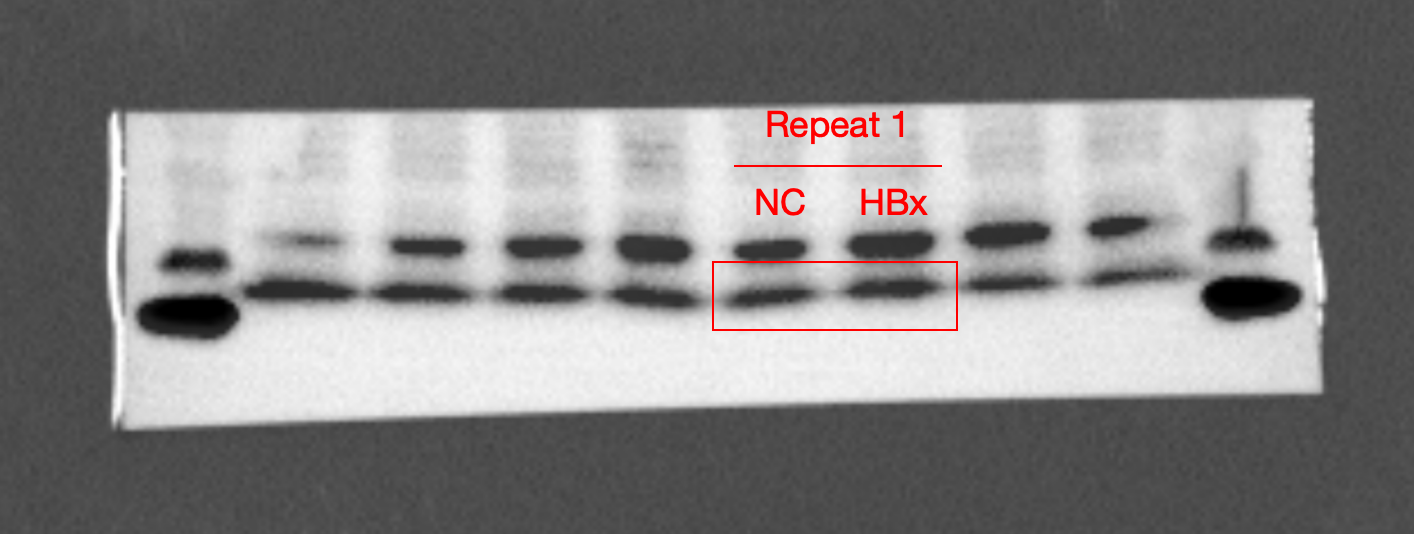

Supplement: S3 Image — (ZIP) [file pone.0325363.s003.zip › Raw_WB_Images/MIF-1 (with annotation).tif]

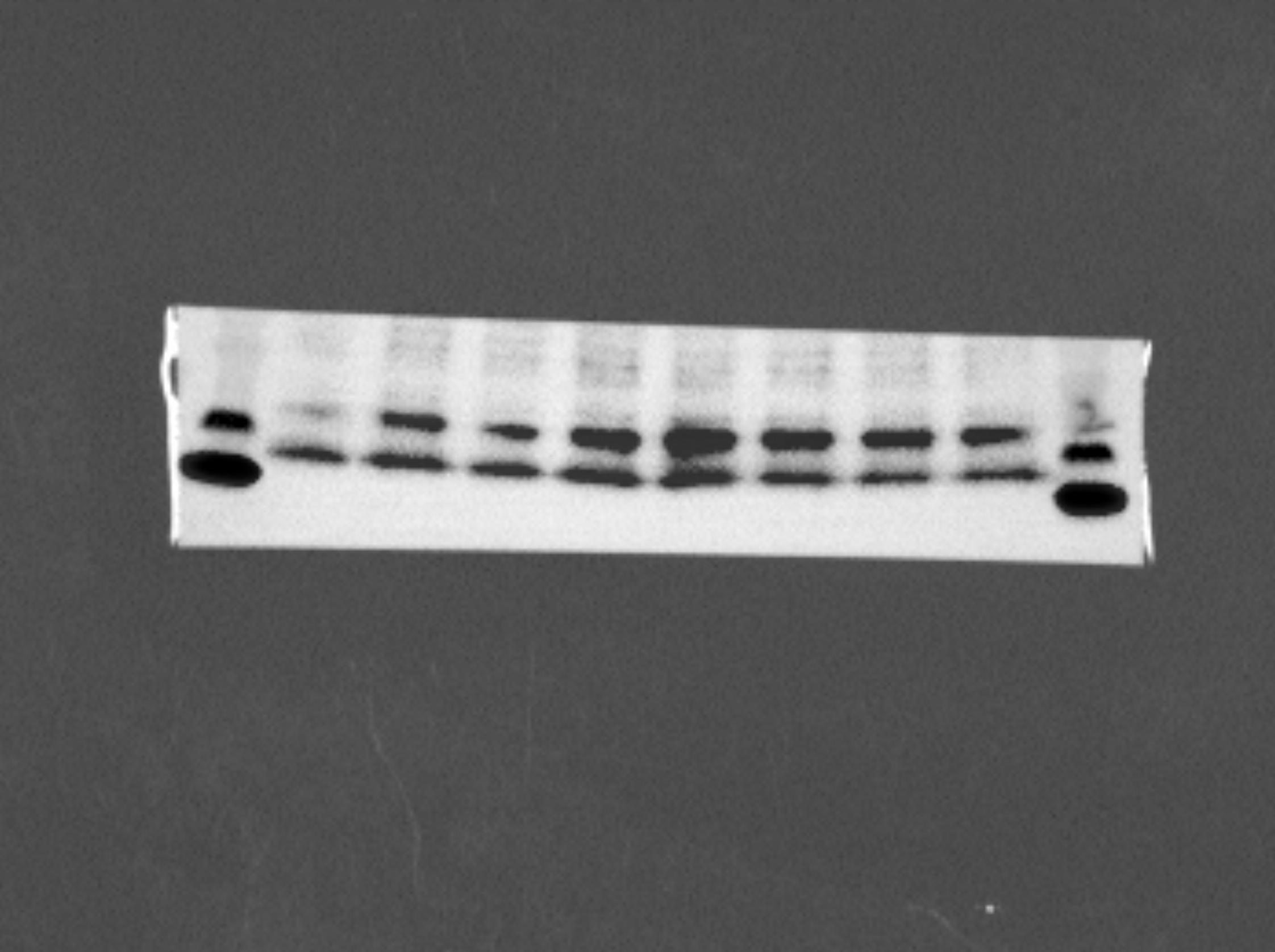

Supplement: S3 Image — (ZIP) [file pone.0325363.s003.zip › Raw_WB_Images/MIF-2.tif]

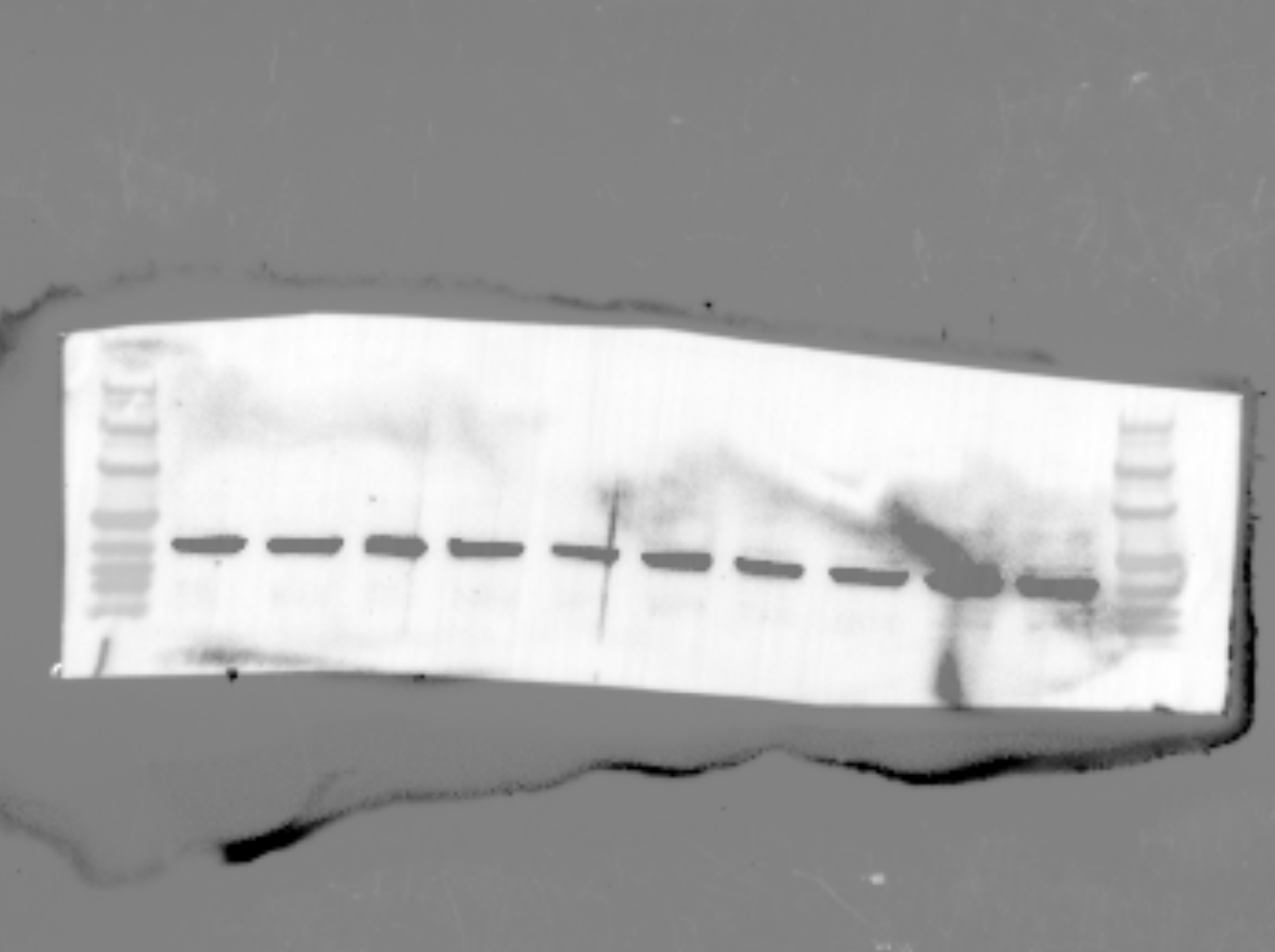

Supplement: S3 Image — (ZIP) [file pone.0325363.s003.zip › Raw_WB_Images/DNTTIP2-tublin-2 (loading control).tif]

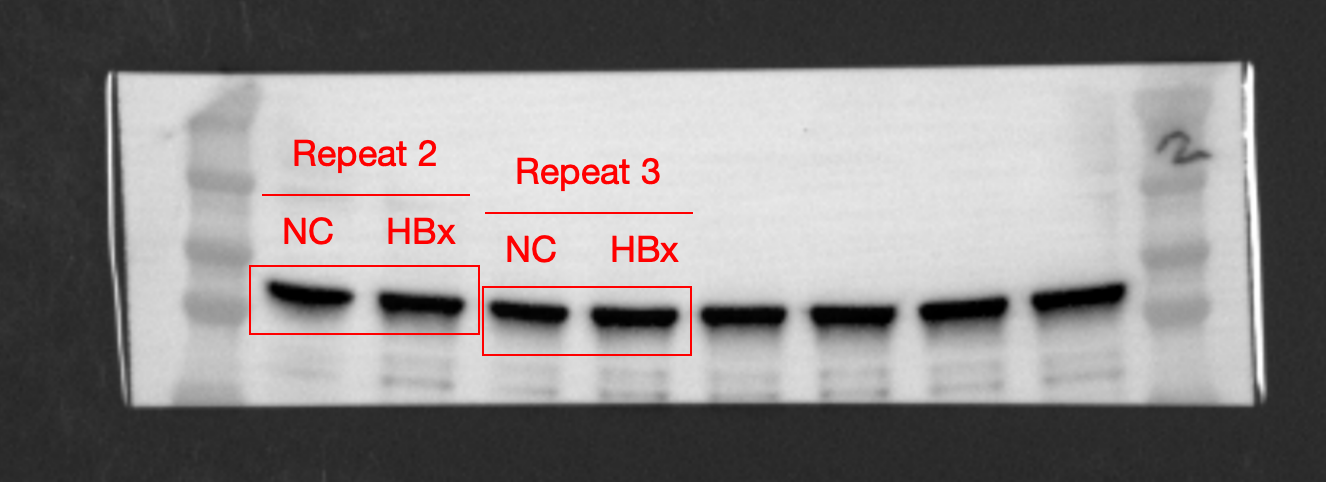

Supplement: S3 Image — (ZIP) [file pone.0325363.s003.zip › Raw_WB_Images/MIF-GAPDH-2 (loading control) (with annotation).tif]

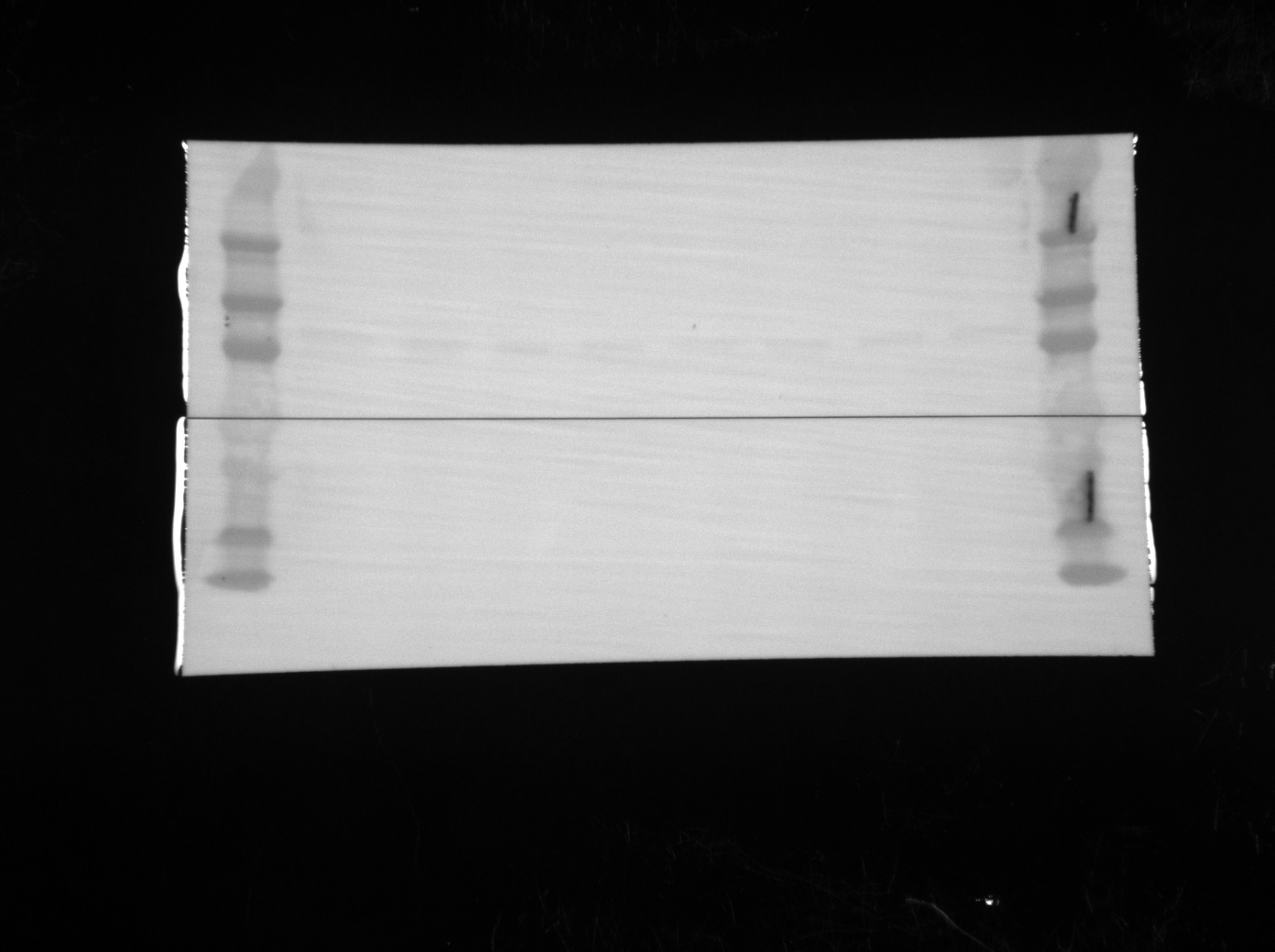

Supplement: S3 Image — (ZIP) [file pone.0325363.s003.zip › Raw_WB_Images/MIF-1 membrane (spliced).tif]

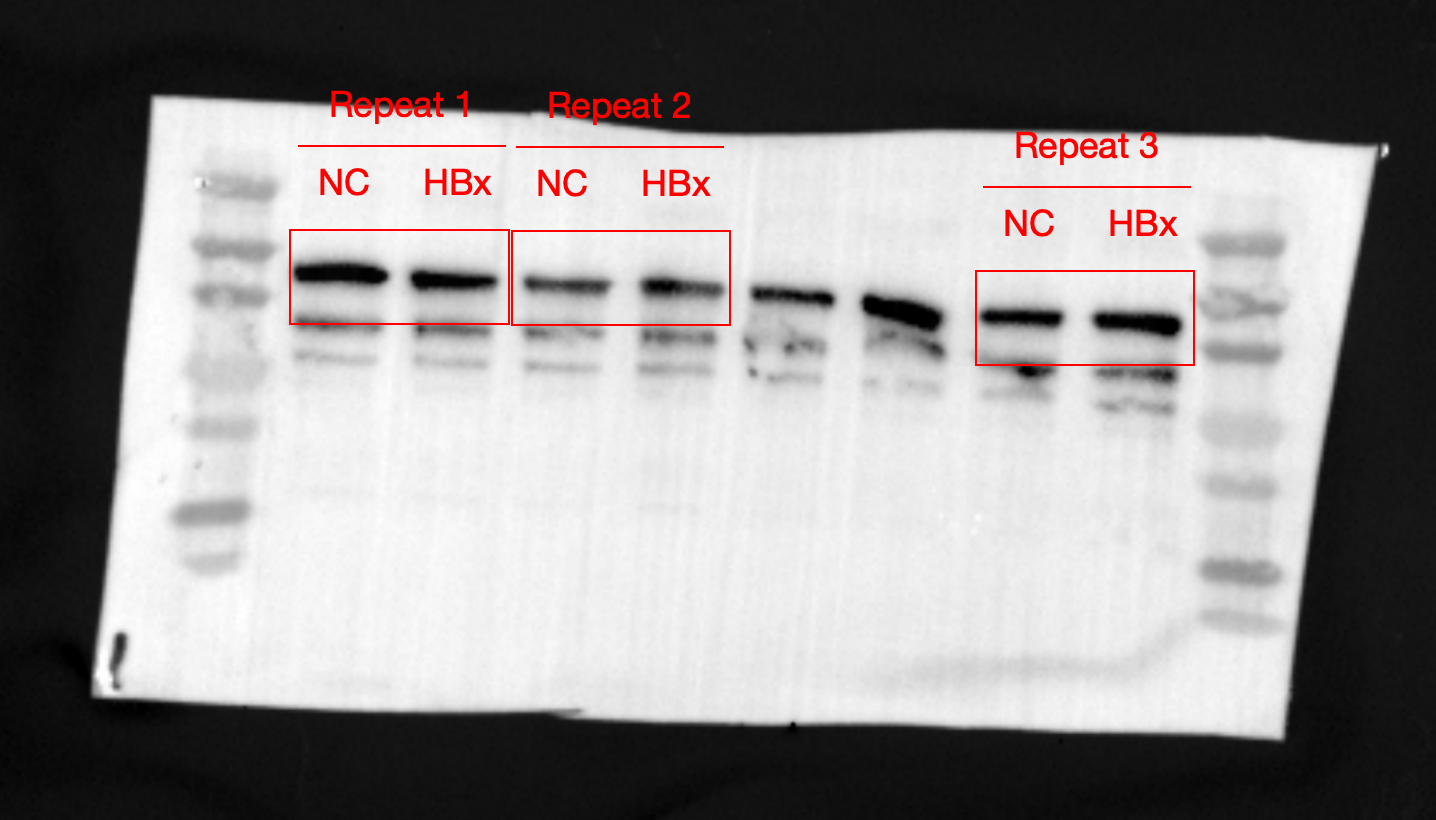

Supplement: S3 Image — (ZIP) [file pone.0325363.s003.zip › Raw_WB_Images/HBx-GAPDH (loading control) (with annotation).tif]

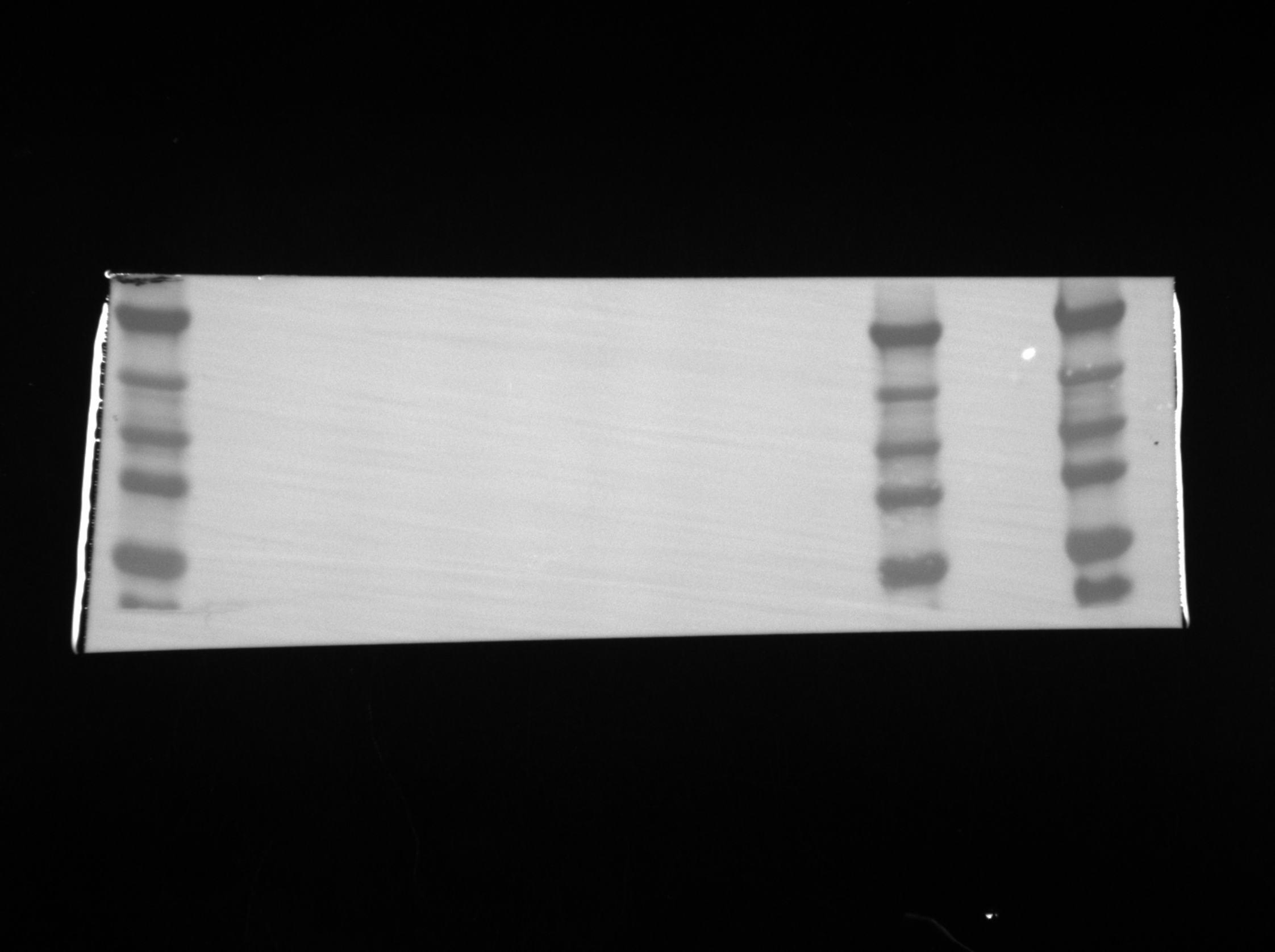

Supplement: S3 Image — (ZIP) [file pone.0325363.s003.zip › Raw_WB_Images/CD74-2 membrane (uncropped).tif]

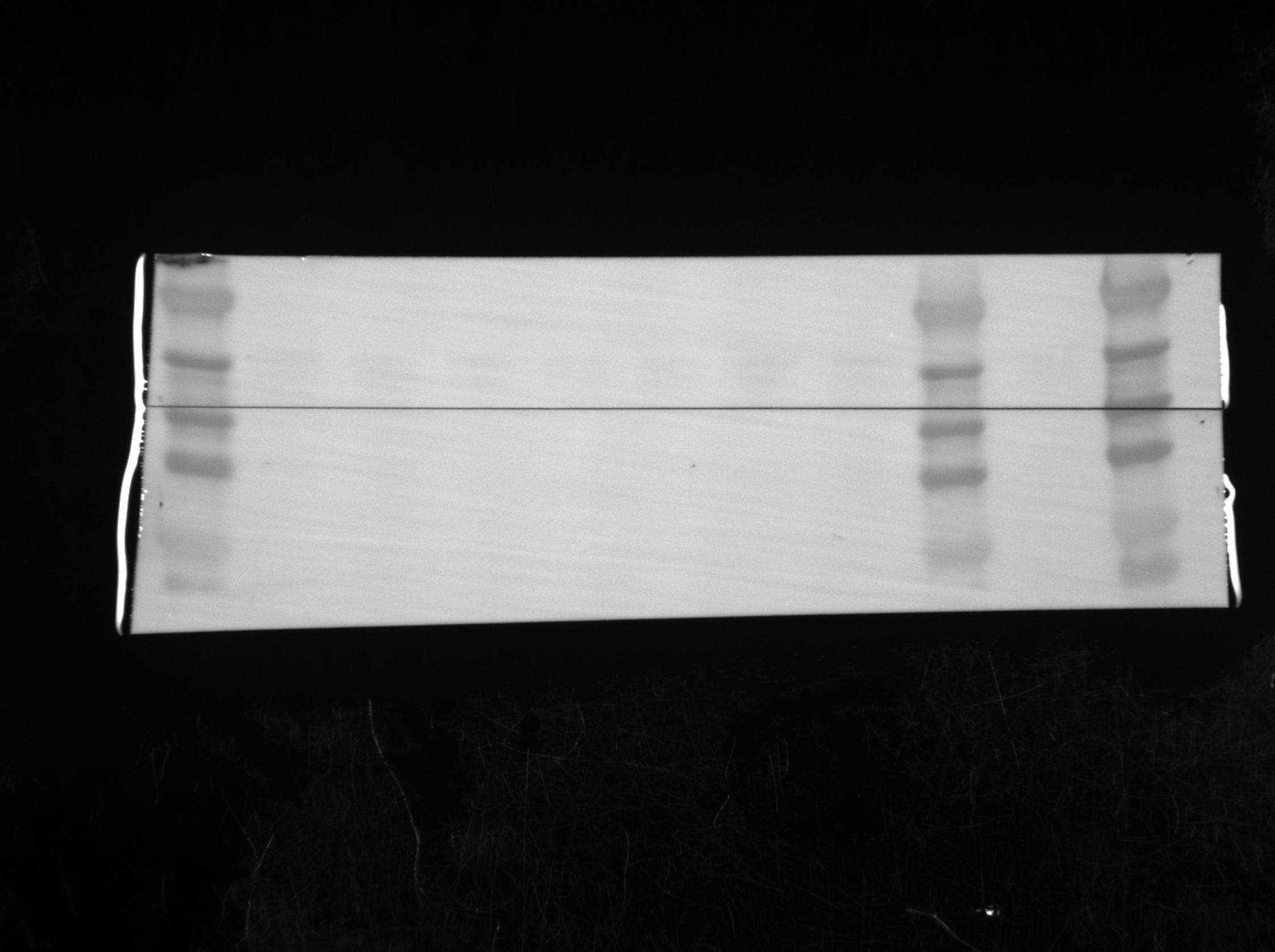

Supplement: S3 Image — (ZIP) [file pone.0325363.s003.zip › Raw_WB_Images/CD74-2 membrane (spliced).tif]
