# Supplementary material for: Hepatitis B virus X protein (HBx)-mediated immune modulation and prognostic model development in hepatocellular carcinoma
Source: PLoS One. 2025 Jun 27;20(6):e0325363. doi: 10.1371/journal.pone.0325363 (PMC12204523; doi:10.1371/journal.pone.0325363)
Supplement: Attachment — Submitted filename: Comments for paper_ PLOS ONE.docx [file pone.0325363.s004.docx]

**Comments for paper**

**Comments to the Authors:**

In this work, Jianhua Zhong and colleagues, used robust bioinformatics analysis and cell experiments to develop a novel HBx-related four-gene prognostic model that may predicts clinical outcomes, immune infiltration, and immunotherapy response to HBV-related HCC. Additionally, their findings suggest that DNTTIP2 is a key regulator of immunosuppression that occurs in the TME, driving HCC tumorigenesis and may serve as a putative prognostic biomarker and therapeutic target for HCC.

Below, I have outlined some points for the authors to address:

1. In the discussion section, the authors should present a more robust account of the canonical function of DNTTIP2 and possibly what is already known about its role in the TME and tumorigenesis. Since experiments to elucidate the molecular mechanism(s) by which DNTTIP2 may drive HCC is lacking.
2. The authors stated that “Between January 2020 and August 2024, HCC tissues from 3 HBV-infected HCC patients and **their corresponding adjacent non-tumor tissues**, as well as HCC tissues from 3 HCC patients without HBV infection and **their corresponding adjacent nontumor tissues**, were collected at the Eighth Affiliated Hospital of Sun Yat-sen University” but representative images of IHC staining of DNTTIP2 in the adjacent non-tumor tumor tissues are not shown. Could the authors provide that?
