# Supplementary material for: Hepatitis B virus X protein (HBx)-mediated immune modulation and prognostic model development in hepatocellular carcinoma
Source: PLoS One. 2025 Jun 27;20(6):e0325363. doi: 10.1371/journal.pone.0325363 (PMC12204523; doi:10.1371/journal.pone.0325363)
Supplement: Attachment — Submitted filename: Response to Reviewers.docx [file pone.0325363.s006.docx]

To the editors,

First of all, we would like to thank the editors for providing us the opportunity to submit a revised version of our manuscript. We would also like to thank the reviewers for their professional remarks and suggestions. We have read through the comments carefully and have made corrections accordingly. All of the questions from the reviewers were answered one by one. The revisions are marked in red, as shown below. We hope this revision can meet the rigorous and professional requirements of *PLOS ONE*.

In addition to addressing the reviewers’ comments, we have made the following revisions in accordance with the journal’s requirements:

1. We have formatted the manuscript according to *PLOS ONE*’s guidelines.
2. We have successfully bound our ORCID iD to the corresponding author’s profile in Editorial Manager.
3. We have provided the original, uncropped, and unadjusted images underlying all blot or gel results as per *PLOS ONE*’s policy.
4. Captions for the Supporting Information files have been included at the end of the manuscript.

We appreciate the constructive feedback provided and believe that these revisions have enhanced the quality of our manuscript.

**Reviewer 1**

On behalf of all the contributing authors, I would like to express our sincere appreciations of your constructive comments. These comments are all valuable and helpful for improving our article. Accordingly, we revised the manuscript. All comments were responded one by one and all the revised points were marked by red. Hope the revision can be satisfactory for the strict and professional publication requirement of *PLOS ONE*.

**Q1: *In the discussion section, the authors should present a more robust account of the canonical function of DNTTIP2 and possibly what is already known about its role in the TME and tumorigenesis. Since experiments to elucidate the molecular mechanism(s) by which DNTTIP2 may drive HCC is lacking.***

**Response:** First of all, we want to express our appreciation for your wise advice and comments. We have expanded the discussion section to provide a more detailed overview of the canonical function of DNTTIP2. We also included what is currently known about its potential involvement in TME and tumorigenesis. According to your comments, the relevant content in the discussion section has been added as follows:

“DNTTIP2, also known as terminal deoxynucleotidyltransferase interacting factor 2 (TdIF2), plays key roles in both cellular processes and tumorigenesis. It is an acidic protein that interacts with TdT and has been identified as a histone chaperone within the nucleus. It binds to DNA and core histones, and its C-terminal region is rich in acidic amino acids, which are important for its function in chromatin dynamics[50]. In the context of cancer, DNTTIP2 has been shown to have a significant role in regulating the cell cycle, particularly in pancreatic cancer (PDAC). Depletion of DNTTIP2 leads to G1 arrest in MIA-PaCa-2 cells and G2 arrest in PK-1 cells, with a corresponding downregulation of cell cycle regulators such as SATB1, CDK6, and CDK1. However, DNTTIP2 depletion does not induce apoptosis, highlighting its more direct role in cell cycle regulation rather than in promoting cell death[51]. Additionally, DNTTIP2 has been shown to influence ribosomal RNA (rRNA) gene transcription by interacting with human ribosomal RNA promoters and promoting hrDNAP activity. Through its interaction with the histone acetyltransferase Tip60, DNTTIP2 inhibits Tip60’s acetylation activity and co-localizes with Tip60 in the nucleolus. This interaction also promotes acetylation of the upstream binding factor (UBF), a key regulator of rRNA synthesis[50]. DNTTIP2 plays a crucial role in both TME and tumorigenesis. It promotes M2 tumor-associated macrophages (TAMs) activation and angiogenesis, processes that contribute to tumor progression. Additionally, DNTTIP2 is involved in the IL6/JAK/STAT3 signaling pathway, which is key to tumor growth and immune evasion. The amplification of DNTTIP2 is associated with poor prognosis in glioma patients, and its hypermethylation is also linked to unfavorable outcomes, highlighting its potential as a biomarker and therapeutic target[52].” (Page 31-32, Lines 627-655)

**Q2: *The authors stated that “Between January 2020 and August 2024, HCC tissues from 3 HBV-infected HCC patients and their corresponding adjacent non-tumor tissues, as well as HCC tissues from 3 HCC patients without HBV infection and their corresponding adjacent nontumor tissues, were collected at the Eighth Affiliated Hospital of Sun Yat-sen University” but representative images of IHC staining of DNTTIP2 in the adjacent non-tumor tumor tissues are not shown. Could the authors provide that?***

**Response:** Thank you for your valuable feedback. We apologize for the omission of representative images of DNTTIP2 IHC staining in the adjacent non-tumor tissues. We have now included these images in the supporting information of the revised manuscript, as the primary focus of the manuscript is on HCC tissues. Our results show that DNTTIP2 is indeed highly expressed in the adjacent non-tumor tissues of HBV-infected patients, similar to its expression in HCC tissues. We have also provided the relevant images below for your reference:

**S2 Fig**. Representative images of H&E staining and IHC staining of DNTTIP2 protein in HBV-negative and HBV-positive adjacent non-tumor tissues.

**Reviewer 2**

On behalf of all the contributing authors, I would like to sincerely thank you for your constructive comments. Your feedback has been invaluable in enhancing the quality of our manuscript. In response, we have revised the manuscript accordingly. Each comment has been addressed individually, and all revisions are highlighted in red. We hope that these changes meet the rigorous and professional standards required by *PLOS ONE*.

**Q1: *Page 17, line 208: Although 2^-ddCt is a common technique, it assumes that the amount of sample is doubled at each cycle, and this is not always the case. It would be convenient to use a standard calibration curve using serial dilutions of a pull of samples. It is also important to check more than one normalizer and use GeNorm or Normfinder to find a normalization factor. This reviewer recommends using 2 or 3 in total or at least demonstrating that GAPDH is a good normalizer by showing the Ct's (which should be fairly similar across samples).***

**Response:** Thank you for your valuable suggestions regarding the normalization methods. We highly appreciate your recommendation to use a standard curve and multiple normalization factors, and we will consider this as a direction for improvement in future studies. Regarding our current qPCR analysis method, our team has been using the 2^-ddCt method. While we plan to explore more advanced normalization techniques in the future, our current method assumes that the amplification efficiency of the target gene and the reference gene is similar. Both the target gene and GAPDH, as the reference gene, exhibit similar slopes during the exponential phase of the amplification curves, indicating consistent amplification efficiency. Although we acknowledge the need for more advanced methods, we are currently able to apply the 2^-ddCt method with reasonable confidence. Regarding GAPDH as a reference gene, we have validated its stability through multiple experiments. GAPDH has been a long-standing and reliable internal control in our lab, and it has been widely used and validated in several relevant studies, particularly in liver cancer and other tumor-related research. We also performed a comparative analysis of the Ct values of GAPDH across different samples, and the results showed consistent expression across all groups, confirming its stability. Additionally, GAPDH’s stability and consistency have been consistently validated under various experimental conditions, further supporting its suitability as a reference gene in our current study. We understand the importance of selecting appropriate normalization factors for ensuring data reliability, and we will continue to refine and validate these methods in future research. We appreciate your insightful feedback, which provides valuable guidance for the direction of our future studies. The table below shows the Ct values of GAPDH across different samples.

|  | the Ct values of GAPDH |  | the Ct values of GAPDH |
| --- | --- | --- | --- |
| NC-1 | 15.01 | HBx-1 | 15.11 |
| NC-2 | 15.04 | HBx-2 | 15.14 |
| NC-3 | 14.22 | HBx-3 | 15.12 |
| NC-4 | 14.17 | HBx-4 | 14.17 |
| NC-5 | 14.13 | HBx-5 | 14.17 |
| NC-6 | 14.15 | HBx-6 | 14.16 |
| NC-7 | 14.38 | HBx-7 | 14.39 |
| NC-8 | 14.44 | HBx-8 | 14.41 |
| NC-9 | 14.42 | HBx-9 | 14.37 |

**Q2: *Page 19, line 267: Here the threshold is set to |logFC|>0.5 (same for figure 1). In the Methods (page 14, line 118) the values were |log2FC|>1, which makes more sense as it assumes a FC greater than 2-fold. Is the value set here log10? Anyway, there is a mistake that needs to be corrected somewhere.***

**Response:** Thank you for your careful review. You are absolutely right — the inconsistency in the fold change threshold was due to a writing error. The correct threshold used throughout our analysis was |log_2_FC| > 0.5, and we have now revised the manuscript to ensure consistency in both the Results and Methods sections. We appreciate you pointing this out. The corresponding content of the manuscript has been modified as follows:

“All identified DEGs met the criteria of P < 0.05 and |log_2_ (Fold-change)| > 0.5. ” (Page 9, Lines 189-190)

“Based on three algorithms, a differential analysis was conducted on HepG2 cells expressing HBx compared to control group (|log_2_FC| > 0.5, P < 0.05). ” (Page 20, Lines 394-396)

**Q3: *Page 20, line 283: FOR ALL THE FIGURES’ FOOTNOTES. Knowing that this is not the final format of the paper, this reviewer suggests for the next submission to put all footnotes together, at the end of the text or with the figures or as supplementary material. It is very difficult to find them when you are reading the text and looking at the figures until you realize that they are at the end of each part.***

**Response:** Thank you for your valuable suggestion regarding the footnotes. We understand that having footnotes scattered throughout the text can make it difficult to follow. In response to your comment, we have now gathered all the footnotes together and placed them at the end of the manuscript for better clarity and ease of reference. We appreciate your feedback and believe this change will improve the readability of the paper.

**Q4: *Page 20, line 287: More information is needed for correct interpretation of Figures 2E-G. The outer boxes, with the dots, are divided into four regions or levels where the dots are drawn. What do those levels mean? The inner boxes, where the z-score color is used, have different sizes. Why?***

**Response:** Thank you for your thoughtful comment regarding the interpretation of Figures 2E-G. We are happy to provide further clarification:

In these figures, the outer boxes represent the distribution of differentially expressed genes in each pathway, based on their log fold change (log_2_FC) values. The red dots indicate upregulated genes, while blue dots indicate downregulated genes. The further a dot is from the center, the greater the absolute log_2_FC value it has. The four regions in each outer box correspond to different log_2_FC intervals, and these intervals are scaled according to the log_2_FC distribution within each individual pathway.

The inner boxes represent the overall activity trend of the pathway based on the z-score. The color intensity reflects the magnitude of the z-score. Additionally, the size of the inner box corresponds to the statistical significance (p-value) of the pathway enrichment - larger boxes indicate more significant pathways.

We have revised the figure legend in the manuscript to explain these visual elements more clearly and help readers interpret the figures correctly:

“**E-G** The enriched GO terms of DEGs: (E) Biological Process, (F) Cellular Component, and (G) Molecular Function. Red and blue dots represent upregulated and downregulated genes based on log_2_FC. Inner box color indicates pathway z-score, and box size reflects enrichment significance.”

**Q5: *Page 20, line 293: Although the text is fine and I would keep it, Figure 3A is too complicated and, in this reviewer's opinion, does not provide much information (Figure 3B is sufficient to understand the results). I suggest moving Figure 3A to supplementary figures.***

**Response:** Thank you for your constructive feedback. We understand your concern regarding the complexity of Figure 3A. After considering your suggestion, we have moved Figure 3A to the supplementary figures section, as Figure 3B alone provides sufficient information for understanding the results. We believe this will enhance the clarity of the main text. Thank you for your helpful input. The revised Figure 3 and the newly added Figure S1 are shown below:

**Fig 3.** PPI Network Analysis, Hub DEGs Identification, and Survival Analysis in HCC.

**A** A key cluster with 12 genes by MCODE based on the PPI network. **B** KM-plot of prognostic HBx-related hub genes based on TCGA cohort. **C** KM-plot of prognostic HBx-related hub genes based on GEPIA. **D** The expression of prognostic hub DEGs in the normal and tumor tissues (***P < 0.001, (****P < 0.001). **E** The correlation between the seven prognostic hub DEGs using Spearman analyses. Positive correlation was marked with red. Colors represent different ranges of Pearson correlation coefficients (e.g., dark red indicates R = 0.8 - 1.0). The colored areas are categorized based on correlation strength, not proportionally scaled to the R value.

**S1 Fig.** Characterization of HBx-Related DEGs and MIF Signaling Interactions in HBV-Related HCC.

**A** PPI network of HBx-related DEGs. **B** The number of interactions and interaction strength between different celltypes in HBV-related HCC tissues. **C** The interaction strength of MIF signaling pathway between different celltypes in HBV-related HCC tissues. **D** Signaling from malignant cells to other cells in the MIF signaling pathway. A thicker line indicates a higher interaction strength or a greater number of interactions.

**Q6: *Page 21, line 305: The upstream transcription factor analysis is a good idea, but these results seem to be no longer used. Does this figure and result add anything to the paper? This reviewer would remove this figure.***

**Response:** Thank you for your valuable comment. After reconsidering the relevance of the upstream transcription factor analysis, we agree that it does not significantly contribute to the main findings of the paper. Therefore, we have removed this figure from the manuscript. The revised Figure 3 is shown above. We appreciate your suggestion, which has helped us streamline the content.

**Q7: *Page 21, line 312: More information is needed for correct interpretation of the 3F figures. The color used in the graph is according to the Pearson R value, but is the colored area also proportional to this value?***

**Response:** Thank you for your comment. The different colored areas in Figure 3F correspond to manually defined ranges of Pearson correlation coefficients (e.g., dark red represents R values between 0.8 and 1). These color assignments are categorical and intended to visually differentiate levels of correlation, but they are not proportionally scaled based on mathematical relationships. We have clarified this in the figure legend accordingly:

“Colors represent different ranges of Pearson correlation coefficients (e.g., dark red indicates R = 0.8 - 1.0). The colored areas are categorized based on correlation strength, not proportionally scaled to the R value.”

**Q8: *Page 21, line 314: Since there is no negative correlation in Figure 3F, the blue color comment is not necessary. I would leave just the red color comment.***

**Response:** Thank you for your suggestion. We agree with your comment and have removed the blue color comment. Only the red color comment, which indicates a high level of positive correlation, has been retained in the revised figure legend:

“Positive correlation was marked with red.”

**Q9: *Page 22, line 336: More info is needed for the correct interpretation of the Figure 4C. What do the columns of the histogram represent?***

**Response:** Thank you for your insightful comment. We have revised the figure legend to clarify that the height of each column in the histogram represents the number of patients falling within specific ranges of the riskscore. The updated legend is as follows:

“**C** The optimal cut-off point to divide riskscore into low and high groups was 5.39294. The height of each column in the histogram represents the number of patients within the corresponding riskscore range.”

**Q10: *Page 22, line 338: The footnote in Figure 4E said “survival,” and that seems to be the same as Figure 4D, and it is not. It would be better to use the same expression from the main text, “mortality risk”.***

**Response:** Thank you for pointing this out. We agree that using consistent terminology improves clarity. We have revised the figure legend for Figure 4E to match the terminology used in the main text. The updated legend now reads:

“**E** The mortality risk in the low-risk and high-risk group patients in the TCGA cohort.”

**Q11: *Page 23, line 364: In Figure 5A, the Y-axis says “expression.” What does that mean? Exactly what is being measured needs to be clarified and explained.***

**Response:** Thank you for your thoughtful comment. We apologize for the lack of clarity. In Figure 5A, the Y-axis represents the enrichment score for each type of immune cell, which reflects the relative abundance of immune cell infiltration in the tissue samples. A higher score indicates a higher level of immune cell infiltration.We have updated the figure legend accordingly to clarify this point:

“**A** Enrichment scores of TME infiltration cells in normal liver versus HCC tissues. Higher scores represent higher levels of immune cell infiltration.”

**Q12: *Page 24, line 374: Again, in Figure 6A, the Y-axis said “expression.” What does that mean? Exactly what is being measured needs to be clarified and explained (on page 25, line 395)***

**Response:** Thank you for your comment. We apologize for the unclear labeling. In Figure 6A, the Y-axis represents the enrichment scores of tumor-infiltrating immune cells, not gene expression levels. These scores reflect the relative abundance of each immune cell type in the tumor microenvironment (TME). A higher enrichment score indicates a higher level of infiltration by that immune cell type. We have clarified this in the revised figure legend:

“**A** Differences in TME infiltration cells between high-risk and low-risk groups. Enrichment scores represent the relative abundance of immune cell infiltration; higher scores indicate greater infiltration levels.”

**Q13: *Page 24, line 375-376: Figures 6B-C compare not only the risk score to those parameters, but also the parameters to each other (the risk score is only one of the variables). If the comparison between the risk score and the other variables is the important information, the “risk score” should be highlighted in the figure. In the Figure 6B, is the size of the dots proportional to the R score or is it indicated just by the color? In Figure 6C, what does the scale bar on the periphery represent? It would be very useful to have an interpretation of the results as is done on page 24, line 380, 383…***

**Response:** Thank you for your insightful comment. We have added clarifications to the figure legend and Results section accordingly. In Figure 6B, the size of each dot is proportional to the absolute value of the risk score (|R|); the larger the dot, the stronger the correlation. In Figure 6C, the scale bar along the periphery indicates the correlation strength represented by the chord width; wider chords reflect higher |R| values. Additionally, to emphasize the role of the risk score, we have made the relevant modifications in the Results section for better interpretation:

“The correlation between the riskscore and immune infiltrating cells was illustrated (Fig 6B), showing varying degrees of association. It further illustrates the relationships between the risk score and key genes involved in immunotherapy and targeted therapy (Fig 6C).” (Pages 24-25, Lines 490-494)

We have also updated the figure legend:

“**B** The correlation between riskScore and TME immune cell types. The size of each dot corresponds to the absolute risk score (|R|), with larger dots indicating stronger correlations. **C** The correlation between riskScore and genes targeted by immunotherapy or targeted therapy. The width of the chords represents the strength of the correlation, with wider connections indicating higher |R| values.”

**Q14: *Page 24, line 387: The bioinformatics analysis that the authors have performed to predict sensitivity to anticancer drugs is very interesting, but it would be much more interesting if these results were also verified in a real experiment. This reviewer suggests using cultures of HepG2 cells (with and without HBx plasmid, as the model used by the authors for other experiments) to test the response of these cells to treatment with these drugs. This would increase confidence in the predictions.***

**Response:** We appreciate the reviewer’s insightful suggestion. In response, we conducted CCK-8 assays using HepG2 cells with and without HBx overexpression to experimentally validate the predicted drug sensitivities. The results confirmed that low-risk group cells (HBx-) exhibited higher sensitivity to sorafenib, while high-risk group cells (HBx+) responded more strongly to lapatinib, erlotinib, and gefitinib. This is consistent with the bioinformatics predictions and strengthens the reliability of our findings. Accordingly, we have added the experimental results to the revised manuscript (Fig 6N) and supplemented the Results section as follows:

**N** Cell viability of HepG2 cells transfected with control (NC) or HBx plasmid after treatment with sorafenib, lapatinib, gefitinib, and erlotinib.

“The results demonstrated that patients in the low-risk group exhibited greater sensitivity to sorafenib, whereas those in the high-risk group were more responsive to lapatinib, erlotinib, and gefitinib (Fig 6M), which was further supported by CCK-8 assay results (Fig 6N).” (Pages 25-26, Lines 513-517)

The added Methods section is shown as follows:

“**Cell Counting Kit-8 (CCK-8) cell viability assay**

Cells transfected with either NC plasmid or HBx plasmid were seeded into 96-well plates at a density of 8000 cells per well, with 100 μL of complete medium. After 12 hours of incubation, the original culture medium was discarded, and fresh complete medium containing different concentrations of sorafenib, lapatinib, gefitinib, and erlotinib (corresponding to the IC50 of each drug) was added. The cells were then incubated for 24 hours. Subsequently, 10 μL of CCK-8 reagent was added to each well, and cell viability was measured by detecting the absorbance at 450 nm using a microplate reader.” (Pages 16-17, Lines 326-335)

**Q15: *Page 25, line 395: See Page 24, line 374 comment.***

**Response:** We appreciate the reviewer’s comment. This question has been addressed in our response to the comment on Page 24, line 374.

**Q16: *Page 27, line 441: “…negative patient, H&E staining…”***

**Response:** Thank you very much for your careful review and valuable suggestions. We have revised the sentence as per your recommendation, changing the period to a comma and adjusting the phrasing for improved clarity and accuracy. We truly appreciate your thoughtful guidance, and we believe these revisions have enhanced the quality of the manuscript. The corresponding content of the manuscript has been revised as follows:

“Furthermore, HCC tissue samples were collected from both HBV-positive and HBV-negative patients, and H&E staining was used to confirm the samples as HCC tissues.” (Pages 28, Lines 555-557)

**Q17: *Page 27, line 446: As I said above, I would put all the footnotes together. The title of Figure 8 needs to be revised. This reviewer would use “Correlation of DNTTIP2 with MIF and verification of DNTTIP2 expression in HBV-related hepatocellular carcinoma.”***

**Response:** We greatly appreciate your insightful suggestion. We have now moved all the footnotes together as you recommended. Additionally, we have revised the title of Figure 8 to “Correlation of DNTTIP2 with MIF and verification of DNTTIP2 expression in HBV-related hepatocellular carcinoma.” We appreciate your input, and hope the revision meets your expectations.

**Q18: *Page 27, line 453: In Figure 8F, since it is sometimes not easy to clearly see the brown color of positive detection, it would be helpful to explain in this text that positivity is marked by that color.***

**Response:** Thank you for your valuable suggestion. We agree that the brown color indicating positive staining may not be clearly distinguishable to all readers. In response, we have carefully reselected and reorganized the representative IHC images in the revised Figure 8E to improve clarity. Additionally, we included immunohistochemical results for MIF and CD74 to support the findings. To further address your comment, we have also added a clarification in the main text stating that “positivity is marked by brown staining,” to help guide the interpretation of the IHC results. The corresponding content of the manuscript has been revised as follows:
“DNTTIP2, MIF and CD74 expression levels in HBV-positive HCC tissues, marked by brown staining, exceeded those in HBV-negative HCC tissues (Fig 8E).” (Page 28, Lines 557-559)

**Reviewer 3**

On behalf of all the contributing authors, I would like to express our sincere appreciations of your constructive comments. These comments are all valuable and helpful for improving our article. Accordingly, we revised the manuscript. All comments were responded one by one and all the revised points were marked by red. Hope the revision can be satisfactory for the strict and professional publication requirement of *PLOS ONE*.

**Q1: *Result 3.2: Seven genes are identified, but from Result 3.3 onwards, only four genes (BRIX1, RPF2, DNTTIP2, and WDR75) are further analyzed. Please include the remaining three genes in the discussion or provide a clear rationale for excluding them.***

**Response:** Thank you for your insightful comment. We performed LASSO regression to construct a predictive model for patient risk. This method introduces a penalty term in the loss function, which causes the coefficients of less important variables to shrink to zero. As a result, only the most predictive features are retained. After applying LASSO regression to the seven genes, three were identified as non-contributory due to their coefficients shrinking to zero and were therefore excluded from further analysis. The relevant explanation has been added at the beginning of Result 3.3 (Construction of Prognostic Riskscore Signature):

“Among the seven prognosis-related genes, four genes with non-zero coefficients were retained by LASSO regression for constructing the riskscore model, while the remaining three were excluded due to minimal predictive contribution.” (Page 22, Lines 436-439)

**Q2:** ***Result 3.4: The latter half of this section describes the relationship between hub genes and specific treatment targets. This content seems unrelated to the title of Result 3.4. I suggest revising the title to better reflect the content or restructuring this part of the manuscript.***

**Response:** Thank you for your thoughtful comment. We agree that the latter part of this section extends beyond immune cell infiltration and includes analysis related to treatment targets. To better reflect the full scope of the content, we have revised the section title to:

“Immune Cell Infiltration and Potential Therapeutic Targets”

We believe this revised title more accurately captures the discussion of both immune landscape and the associations between hub genes and specific therapeutic targets.

**Q3: *Results 3.6-3.7: The manuscript suggests that the MIF signaling pathway is crucial for the development of the TME in HBV-related HCC and that DNTTIP2 may modulate this pathway to induce immunosuppression. High expression of DNTTIP2 is validated in HepG2 cells and HCC tissue samples. However, the role of the MIF signaling pathway itself has not been experimentally validated. It is recommended to test key molecules in the MIF signaling pathway, such as MIF and CD74, in HepG2 cells and human HCC samples to further substantiate these findings.***

**Respond:** Thank you for your thoughtful and constructive feedback. We appreciate your suggestion to experimentally validate the role of the MIF signaling pathway in the context of HBV-related HCC. In response, we have now tested the expression levels of key molecules in the MIF signaling pathway, including MIF and CD74, in both HBx-overexpressing HepG2 cells and HBV-positive HCC tissue samples. Our results show that the expression of MIF and CD74 is indeed elevated, which is consistent with the high expression of DNTTIP2. These findings further support our hypothesis that DNTTIP2 may modulate the MIF signaling pathway in the development of immunosuppression in the TME of HBV-related HCC. We have included these new data in the revised manuscript and hope this addresses your concerns. Thank you once again for your valuable suggestion. The relevant content has been added in the manuscript:

“The result of qPCR and Western blot analysis indicated that DNTTIP2 mRNA and protein expression was significantly upregulated in HepG2 cells expressing HBx (Fig 8C, D). Moreover, we examined the expression of MIF and CD74, key molecules in the MIF signaling pathway, and found that their protein levels were also elevated in HBx-overexpressing HepG2 cells (Fig 8D). Furthermore, HCC tissue samples were collected from both HBV-positive and HBV-negative patients, and H&E staining was used to confirm the samples as HCC tissues (Fig 8E). DNTTIP2, MIF and CD74 expression levels in HBV-positive HCC tissues, marked by brown staining, exceeded those in HBV-negative HCC tissues (Fig 8E). ” (Pages 27-28, Lines 549-559)

**Fig 8.** Correlation of DNTTIP2 with MIF and verification of DNTTIP2, MIF and CD74 expression in HBV-related hepatocellular carcinoma.

**A** Gene expression levels of DNTTIP2 and MIF in GSE202642 dataset. **B** Correlation between DNTTIP2 and MIF expression based on TCGA cohort. **C** Expression of DNTTIP2 mRNA in HepG2 cells transfected with HBx plasmid or control plasmid (NC). **D** Expression of DNTTIP2, CD74 and MIF protein after transfection with HBx plasmid and control plasmid (NC) in HepG2 cells. **E** Representative images of H&E staining and IHC staining of HBV-negative and HBV-positive HCC tissue. IHC staining was performed to detect DNTTIP2, MIF, and CD74 expression. Brown staining indicates positive expression. Scale bars, 100 μm and 20 μm. Data presented as mean ± SEM (n = 3). *P < 0.05; **P < 0.01; ***P < 0.001; ****P < 0.0001.

**Q4: *Functional Validation: While the manuscript validates high expression of DNTTIP2 in HBx-expressing HepG2 cells through qPCR and Western blotting, it lacks direct in vivo functional validation. It is recommended to conduct experiments in an HCC mouse model by knocking down or overexpressing DNTTIP2, observing its effects on tumor growth, immune cell infiltration, and response to immunotherapy, to further validate its functional role in the TME.***

**Respond:** Thank you for your insightful suggestion regarding in vivo functional validation. We fully acknowledge the importance of investigating DNTTIP2’s role in an HCC mouse model, as it would provide more comprehensive evidence of its impact on tumor growth, immune cell infiltration, and response to immunotherapy. Unfortunately, due to current practical constraints, including limited resources, time limitations, and the challenges associated with establishing an appropriate in vivo model, we are unable to conduct these experiments at this stage. We believe that the bioinformatics analysis and the in vitro experiments we have conducted so far provide initial evidence supporting the role of DNTTIP2 in HCC. These preliminary findings have helped us understand its potential involvement in the tumor microenvironment, but we acknowledge that further validation through animal models will be essential for a more comprehensive understanding. We deeply regret that we are unable to incorporate this aspect of the study at this time. We sincerely appreciate your constructive feedback and fully recognize that this is a limitation of our current work. We are committed to addressing this gap in our future research by incorporating animal models to further explore the functional role of DNTTIP2 in the tumor microenvironment.

**Q5: *Introduction: In the third paragraph of the introduction, a more comprehensive review of the related literature would strengthen the discussion. For instance, the following studies should be referenced and cited: PMID: 36939783, PMID: 39368944, etc. A more thorough overview of current research and treatment for HBV should be provided.***

**Respond:** Thank you for your valuable suggestion. We have carefully considered your recommendation and have incorporated the requested references into the third paragraph of the introduction. We believe these additions provide a more thorough overview of current research and treatment for HBV, as they address recent advancements in genetic factors and potential antiviral therapies related to HBV infection and its associated complications. Thank you again for your helpful recommendation. Below is the added portion to the revised paragraph:

“In this regard, recent studies have highlighted the potential impact of genetic polymorphisms, such as the ALDH2 Glu504Lys mutation, in shaping the susceptibility to HBV-related diseases and influencing the immune response in East Asian populations[26]. Furthermore, natural product-based antiviral candidates, such as baicalein and its glucuronide derivative baicalin, have gained attention for their antiviral properties and may offer promising therapeutic strategies for HBV-related diseases and HCC[27].” (Page 6-7, Lines 122-130)

Supplementary references:

26.	X Zhang, A Sun, and J Ge, Origin and Spread of the ALDH2 Glu504Lys Allele. Phenomics, 2021. 1(5): p. 222-228.

27.	X Y Liu, W Xie, H Y Zhou, H Q Zhang, and Y S Jin, A comprehensive overview on antiviral effects of baicalein and its glucuronide derivative baicalin. J Integr Med, 2024. 22(6): p. 621-636.

**Q6: *Minor Grammatical Issues: There are some minor grammatical errors in the manuscript. For example, the sentence: "Immune analysis revealed that high-risk patients had lower survival rates, reduced anti-tumor immune cell infiltration, poorer immunotherapy responses, and increased immune evasion." could be rephrased for clarity. Consider revising it to: "Immune analysis revealed that high-risk patients exhibited lower survival rates, decreased infiltration of anti-tumor immune cells, poorer responses to immunotherapy, and increased immune evasion."***

**Respond:** Thank you for pointing out the minor grammatical issues. We have revised the sentence as suggested for improved clarity and streamlined it: “High-risk patients exhibited lower survival rates, decreased infiltration of anti-tumor immune cells, poorer responses to immunotherapy, and increased immune evasion.” Additionally, we have thoroughly reviewed the manuscript and addressed any grammatical issues throughout the text. We appreciate your careful review and believe these revisions enhance the clarity and quality of the manuscript.

**Reviewer 4**

On behalf of all the contributing authors, I would like to sincerely thank you for your constructive feedback. Your comments have been incredibly valuable and have greatly contributed to improving our manuscript. In response, we have revised the manuscript accordingly. Each comment has been addressed individually, with all changes highlighted in red. We hope that the revisions meet the rigorous standards and professional requirements of *PLOS ONE*.

**Q1: *The most important one is undoubtedly the problem of figure resolution. Most of them if not all of them are not exploitable for an honest and benevolent observer.***

**Response:** Thank you for bringing up the issue of figure resolution. We would like to clarify that we actually uploaded high-resolution TIFF images generated using Adobe Illustrator. However, we are sorry to hear that they may not have been displayed clearly, possibly due to some unknown reasons during the submission or viewing process. To ensure clarity and resolution, we have re-uploaded high-quality TIFF images. We sincerely apologize for any inconvenience caused by the figure resolution issue and greatly appreciate your understanding and constructive comments.

**Q2: *The second problem concern some inferences stemming from in silico analyses that are not always clearly explained or transparent.***

**Response:** We appreciate the reviewer’s feedback regarding the in silico analyses. We acknowledge that some of the inferences drawn from these analyses were not clearly explained. To improve clarity, we will provide a more detailed description of the methodology, including the specific computational tools and databases used, as well as the criteria for interpreting the results. We will also highlight any assumptions made during the analysis. In addition, we will ensure that all findings derived from in silico methods are presented with sufficient context to facilitate transparency. The specific revisions will be addressed in detail in the following questions.

**Q3: *Abstract: "Immune analysis revealed that high-risk patients had lower survival rates" this is a pleonastic statement.***

**Response:** Thank you for your insightful suggestion. We understand the concern regarding potential redundancy in this statement. To improve clarity and conciseness, we have revised the sentence as follows:

“High-risk patients exhibited lower survival rates, decreased infiltration of anti-tumor immune cells, poorer responses to immunotherapy, and increased immune evasion.” (Page 2, Lines 38-40)

**Q4: *Was MIF expression also correlated with that of DNTTIP2 in bulk RNA seq data?***

**Response:** We thank the reviewer for this insightful question. In the TCGA-LIHC bulk RNA sequencing dataset, we observed a positive correlation between MIF and DNTTIP2 expression (Figure 8B). These findings indicate a significant association between the two genes in liver cancer samples. The relevant content is shown below:

“Correlation analysis demonstrated that MIF expression has the strongest association with DNTTIP2 expression among key molecules (Fig 8B).” (Page 27, Lines 544-546)

**Q5: *Were all 374 HCC analyzed obtained from HBV-infected patients?***

**Response:** We thank the reviewer for raising this important question. According to the clinical information available in the TCGA-LIHC dataset, viral infection status is not provided, so we are unable to confirm whether all 374 HCC samples were obtained from HBV-infected patients. We have acknowledged and discussed this point in the Discussion section of the revised manuscript:

“Previous studies have developed HCC prognostic models based on cuproptosis, mitochondrial unfolded protein responses, and other gene signatures[39, 40]. However, no prior investigations have specifically focused on HBx-related genes. Our study addressed this gap by identifying DEGs associated with HBx-expressing HCC and establishing a novel prognostic model incorporating four key HBx-related hub genes. Unlike previous approaches, our model highlights the specific impact of HBx on HCC prognosis and tumor immunity, offering new insights into the unique role of HBx in shaping the tumor microenvironment. Although the TCGA-LIHC dataset used for model construction does not contain information on HBV infection status - making it unclear whether all 374 HCC samples were from HBV-infected patients - the integration of HBx-associated genes ensures the model’s relevance to HBV-related HCC. This model not only stratifies patients into low- and high-risk groups with distinct survival outcomes but also predicts responses to immunotherapy, demonstrating its potential clinical utility.” (Page 29, Lines 579-594)

**Q6: *Single-cell transcriptome: Mention that it comes from publicly available data and provide the accession number.***

**Response:** We thank the reviewer for this suggestion. The single-cell transcriptome data used in this study comes from publicly available data (GSE202642). The corresponding accession number has been included in the revised manuscript for reference:

“The single-cell transcriptome data was obtained from the GEO database[28] (GSE202642), including 7 HBV-related HCC samples and 4 adjacent liver tissue samples.” (Page 9, Lines 184-186)

**Q7: *Workflow: HepG2 does not appears on the workflow. It is difficult to understand its importance in the whole analytic process.***

**Response:** We thank the reviewer for this comment. HepG2 cells are an important and widely accepted liver cancer cell line, commonly used in our laboratory and many other studies for in vitro experiments and analyses related to hepatocellular carcinoma (HCC) research. Although we did not explicitly mention HepG2 cells in the workflow, we referred to experimental validation for the sake of conciseness and clarity. This does not affect the fact that HepG2 cells are indispensable in the experimental process.

**Q8: *Furthermore it is usure that these experiments on HepG2 are validating exvivo data or if they are simply relevant. We do not know the expression status of the HBx hub genes HepG2.***

**Response:** Thank you for your comment. In response, we would like to clarify that the validation performed in HepG2 cells is not merely indicative of relevance. We have conducted comprehensive validation of the HBx hub genes at the nucleic acid, protein, and clinical levels. HepG2 cells are widely used in liver cancer research and have been a long-established model in our lab. HepG2 cells themselves do not express HBx and are used to represent HBV-negative HCC in vitro. To model HBV-positive HCC, we transfect HepG2 cells with the HBx plasmid. In our experiments, DNTTIP2, as a hub gene, was highly expressed in HBx-transfected HepG2 cells, and its expression in HBV-positive HCC tissues was also higher than in HBV-negative tissues. This supports the reliability of HepG2 as a model for studying HBV-related HCC. Thank you again for your insightful comment. We appreciate your careful review and are grateful for the opportunity to clarify the use of the HepG2 model in our study.

**Q9: *Why doing LASSO Cox regression of four genes only and not on all hub genes?***

**Response:** We thank the reviewer for this question. In our study, we performed LASSO Cox regression on all seven hub genes and, after the analysis, excluded three genes that were deemed unimportant. LASSO regression is a feature selection method that adds a penalty term to the loss function, shrinking the coefficients of less important variables to zero. As a result, only the genes with the greatest predictive ability for the outcome were retained. After applying this method, the remaining four genes were considered the most relevant for predicting patient risk or disease progression, while the other three were excluded.

**Q10: *Immune cell infiltration: Figures 5A, C, D are not relevant to this paper.***

**Response:** We appreciate the reviewer’s feedback regarding Figures 5A, C, and D. However, we respectfully disagree with the assessment that these figures are not relevant to the paper. In this study, we examine the role of immune cell infiltration in the tumor microenvironment (TME) and its association with hub genes. Figures 5A, C, and D provide critical insights into immune cell infiltration patterns in HCC tissues, which are essential for understanding the relationship between hub genes and immune cell dynamics within the TME. Specifically, Figure 5A shows distinct differences in immune cell infiltration between normal and tumor samples, Figure 5C highlights the reduced immune and stromal activity in HCC tissues, and Figure 5D further supports these findings by showing the associated immune changes. These results are integral to understanding how hub genes may influence immune responses and contribute to tumor progression. Therefore, we believe these figures are essential to the overall narrative of the manuscript.

**Q11: *Figure 5B: it is difficult to see the negative correlation. It seems that there is a lack of any correlation. Please provide the name of cell categories suspected to promote tumor progression and that positively correlated with the 4 hub gene expression. Are CD4 lymphocytes included?***

**Response:** We thank the reviewer for the comment. Regarding Figure 5B, we would like to clarify that the color scheme used indicates positive correlations in red and negative correlations in blue, with deeper colors representing stronger correlations. We have now added the names of the cell categories that are positively correlated with the expression of the four hub genes and are suspected to promote tumor progression in the revised manuscript. Notably, CD4 lymphocytes are included among these cell types. These details have been added in the Results section:

“Furthermore, the expression of hub genes demonstrated a negative correlation with anti-tumor cells, including NK cells, Th1 cells, and effector memory CD8+ T cells. At the same time, it exhibited a positive correlation with cells that may promote tumor progression, such as activated CD4+ T cells, dendritic cells, NK T cells, central memory CD4+ T cells, effector memeory CD4+ T cells and Th2 cells (Fig 5B). ” (Pages 23-24, Lines 470-476)

**Q12: *RPF2 display an inverse correlation with VEGFR: this is somewhat counterintuitive for a gene decreasing survival time for HCC patients. It is absolutely not commented. Why?***

**Response:** We thank the reviewer for pointing this out. Indeed, the negative correlation between RPF2 expression and VEGFR expression may seem counterintuitive, particularly since RPF2 is associated with a decrease in survival time for HCC patients. However, we would like to clarify that this correlation does not imply a direct causal relationship, where an increase in RPF2 expression leads to a decrease in VEGFR expression. Instead, it suggests that RPF2 may promote tumor progression through alternative pathways, independent of VEGFR. The primary aim of showing this correlation is to highlight potential treatment targets. Specifically, our findings suggest that HCC patients with high RPF2 expression may be more inclined to benefit from therapies targeting pathways other than VEGFR. We added this explanation in the discussion section of the revised manuscript for better clarity:

“Interestingly, in our study, we observed a negative correlation between RPF2 expression and VEGFR expression. While this may seem counterintuitive, especially considering RPF2’s role in promoting liver cancer progression, this correlation does not imply a direct causal relationship. It rather suggests that RPF2 may promote tumor progression through alternative pathways independent of VEGFR, highlighting the potential for targeting different pathways in HCC treatment.” (Pages 30-31, Lines 616-623)

**Q13: *Figure 6A is not obvious as there is a large overlap between high risk and low risk groups.***

**Response:** We thank the reviewer for the comment. Regarding Figure 6A, we acknowledge that there is some overlap between the high-risk and low-risk groups. This overlap is expected, as immune infiltration in the TME is influenced by various factors, and the differences between the groups are not always pronounced. As stated in the conclusion, the differences in immune infiltration between the high-risk and low-risk groups were not statistically significant (ns) for some cell types. For those cell types where significant differences were observed, the overlap is minimal, and the differences are clearly marked with asterisks at the top of the figure.

**Q14: *Figure 6B seems to be a correlation matrix between cells population. We do not see where is the risk score exactly.***

**Response:** Thank you for your comment. Figure 6B indeed illustrates the correlation between the risk score and immune cell populations in the TME. Each point on the plot represents the risk score’s correlation with a specific immune cell population. The size of the dots corresponds to the absolute value of the risk score (|R|), with larger dots indicating stronger correlations. The color of the dots reflects the direction of the correlation: red indicates a positive correlation, while blue indicates a negative correlation. We have revised the figure legend to further clarify this explanation for better understanding:

“**B** The correlation between riskScore and TME immune cell types. The size of each dot corresponds to the absolute risk score (|R|), with larger dots indicating stronger correlations.”

**Q15: *Figure 6HK: all IPS scores are lower in high-risk tumors even fucntionnally opposite scores such as CTLA4+/PD1+ and CTLA4-/PD1-. What should we conclude? It is very confusing. In general this series of figure are very difficult to understand with the minimalist legend provided.***

**Response:** Thank you for your valuable feedback. The Immunophenoscore (IPS) is a computational metric where higher scores indicate stronger tumor immunogenicity and a greater likelihood of response to immune checkpoint inhibitors (ICIs). In our analysis (Figure 6H-K), all IPS scores, including CTLA4+/PD1+, CTLA4+/PD1-, CTLA4-/PD1+, and CTLA4-/PD1-, are consistently lower in the high-risk group compared to the low-risk group. This suggests that high-risk tumors are generally less immunogenic and less likely to benefit from immunotherapy, while the low-risk group may have better responsiveness to ICIs.

Regarding the interpretation of functionally opposite combinations (e.g., CTLA4+/PD1+ vs. CTLA4-/PD1-), these scores do not necessarily exhibit inverse trends. Each score reflects a specific hypothetical treatment response scenario, and it is not uncommon for their trends to be aligned, as seen in previous studies such as PMID: 37461041.

To improve clarity, we have revised the figure legend to provide a more detailed explanation of the IPS scoring system and its interpretation in the context of immunotherapy response:

“The IPS scores for CTLA(+)PD1(-), CTLA(-)PD1(+), CTLA(+)PD1(+), and CTLA(-)PD1(-) were all significantly lower in the high-risk group (Fig 6H-K). These findings suggest that patients in the high-risk group may exhibit lower tumor immunogenicity and reduced responsiveness to immune checkpoint inhibitors, including anti-PD1 and anti-CTLA4 therapies.” (Page 25, Lines 501-506)

“**H-K** IPS scores of PD1 and CTLA4 in high-risk and low-risk groups. Low IPS scores in high-risk tumors suggest poor response to immune checkpoint inhibitors, while higher IPS scores in low-risk tumors suggest a better response. (H) Negative response to PD1 treatment and CTLA4 treatment [CTLA(-)PD1(-)]. (I) Positive response to PD1 treatment and negative response to CTLA4 treatment [CTLA(-)PD1(+)]. (J) Negative response to PD1 treatment and positive response to CTLA4 treatment [CTLA(+)PD1(-)]. (K) Positive response to PD1 treatment and CTLA4 treatment [CTLA(+)PD1(+)].”

**Q16: *How many patients have been studied by ScRNA-seq?***

**Response:** Thank you for your question. The scRNA-seq data used in this study is from the publicly available dataset GSE202642, which includes sequencing data from liver cancer tissues related to HBV from 7 patients.

**Q17: *The cell chat figure is not explained. It looks as a PCA analysis but we hardly see any “communication” between cell populations.***

**Response:** Thank you for your feedback. We apologize for the lack of explanation regarding the CellChat figure. To clarify, we have added Figure S1 to further elaborate on the cell-cell interactions. As indicated in the figure legend:

Figure S1B shows the number of interactions and interaction strength between different cell types in HBV-related HCC tissues.

Figure S1C demonstrates the interaction strength of the MIF signaling pathway between different cell types in these tissues.

Figure S1D highlights signaling from malignant cells to other cells within the MIF signaling pathway.

This additional information should help clarify the communication between cell populations and the strength of their interactions, particularly within the context of the MIF signaling pathway. Figure S1 is shown below:

**S1 Fig.** Characterization of HBx-Related DEGs and MIF Signaling Interactions in HBV-Related HCC.

**A** PPI network of HBx-related DEGs. **B** The number of interactions and interaction strength between different celltypes in HBV-related HCC tissues. **C** The interaction strength of MIF signaling pathway between different celltypes in HBV-related HCC tissues. **D** Signaling from malignant cells to other cells in the MIF signaling pathway. A thicker line indicates a higher interaction strength or a greater number of interactions.

**Q18: *Honestly, the impact of HBx on protein staining in H&E is quite difficult to define (increase? Decrease?)***

**Response:** Thank you for your insightful comment. We agree that H&E staining primarily provides morphological information and is not suitable for assessing the expression levels of specific proteins. To better illustrate the differences in protein expression influenced by HBx, we have supplemented our study with immunohistochemical (IHC) staining for DNTTIP2, MIF, and CD74 in HBV-positive and HBV-negative HCC tissues. These markers, indicated by brown staining, provide clearer evidence of protein expression changes, which cannot be reliably interpreted from H&E staining alone. We have updated the figure and revised the text accordingly to reflect this improvement:

“DNTTIP2, MIF and CD74 expression levels in HBV-positive HCC tissues, marked by brown staining, exceeded those in HBV-negative HCC tissues (Fig 8E).” (Page 28, Lines 557-559)

**Reviewer 5**

On behalf of all the authors, I would like to express our sincere gratitude for your thoughtful and constructive feedback. Your comments have been extremely helpful and have significantly improved the quality of our manuscript. In response, we have carefully revised the manuscript, addressing each comment individually, with all changes marked in red. We hope that these revisions align with the high standards and professional requirements of *PLOS ONE*.

**Q1: *The manuscript uses GSE276530 as the primary dataset for gene selection, yet it lacks background information regarding this dataset. Moreover, the specific cell treatment processes and experimental methods are not detailed, which raises concerns about the reliability of the data.***

**Response:** Thank you for your valuable comment. We have added background information regarding the GSE276530 dataset to provide context for its use in gene selection. Additionally, we have included more detailed descriptions of the cell treatment processes and experimental methods to ensure the reliability and reproducibility of the data. We believe these additions enhance the clarity and robustness of the manuscript. The corresponding content of the manuscript has been revised as follows:

“**Background information**: The dataset GSE276530 consists of RNA sequencing data from six HepG2 cell samples, with three transfected with control plasmids and three transfected with HBx plasmids. The purpose of this experiment was to identify differentially expressed genes in HepG2 cells overexpressing HBx, in order to further analyze genes associated with poor prognosis in HBV-related HCC. HepG2 cells serve as a representative model for liver cancer research, and HBx is a key protein produced by HBV. The differential gene expression data obtained from this study will aid in understanding the molecular mechanisms underlying the poor prognosis of HBV-infected HCC patients.” (Page 9, Lines 171-180)

“**Cell culture and transfection**: HepG2 cells were cultured in DMEM medium containing 10% fetal bovine serum and 1% penicillin-streptomycin, and incubated at 37℃ in a humidified atmosphere with 5% CO2. Cells were digested with trypsin and seeded into well plates. On the following day, when the cells reached 60-70% confluence, the medium was replaced with serum-free medium. According to the instructions for Lipofectamine 2000 (Invitrogen, USA), the transfection plasmids were diluted separately in serum-free medium. The cells were incubated at 37℃ for 5 hours, after which the medium was replaced with complete medium and incubation continued until further experiments.” (Page 8, Lines 152-162)

“**Western blotting**: 72 hours after cell transfection, cells were lysed at 4℃ using RIPA buffer containing PMSF. After 30 minutes, the lysates were collected into 1.5 mL EP tubes. The cells were further lysed by sonication (5 seconds on, 5 seconds off) for a total of 3 minutes. The lysates were then centrifuged at 12,000 rpm for 10 minutes at 4℃, and the supernatant was transferred to new EP tubes. Protein concentrations were determined using a BCA protein assay kit (Beyotime Biotechnology), and equalized across groups. 6× loading buffer was added at the appropriate ratio, and the samples were heated in a metal bath at 100℃ for 10 minutes before being stored at -80℃ for further use. Protein samples were taken out from the -80℃ freezer and heated in a metal bath at 100℃ for 10 minutes. After a brief centrifugation and mixing, the samples were loaded into the wells of an SDS-PAGE gel. Electrophoresis was performed at a constant voltage, starting at 80V and then switching to 120V. The PVDF membrane was soaked in methanol for 10 minutes and transferred using wet transfer at 300 mA in an ice bath. After transfer, the membrane was blocked with 5% skim milk at 37℃ on a shaker for 2 hours. The membranes were incubated with the following primary antibodies overnight at 4℃: anti-β-tubulin (Cell Signaling Technology, β-Tubulin (9F3) Rabbit mAb #2128, 1:1000), anti-GAPDH (Cell Signaling Technology, GAPDH (D16H11) Rabbit mAb #5174, 1:1000), anti-HBx (Abcam, Recombinant Anti-Hepatitis B Virus protein X antibody [EPR27041-49] (ab309352), 1:1000), anti-DNTTIP2 (ThermoFisher Scientific, TDIF2 Polyclonal Antibody, PA5-60731, 1:1000), anti-MIF (Cell Signaling Technology, MIF (E7T1W) Rabbit mAb #87501, 1:1000) and anti-CD74 (Servicebio, CD74 Rabbit pAb #GB115427, 1:1000). The next day, the membrane was washed five times with TBST and once with TBS, each for 5 minutes. It was then incubated with secondary antibody on a shaker at room temperature for 45 minutes [Horseradish peroxidase (HRP)-conjugated anti-rabbit IgG antibody (Zhongshan Golden Bridge Biotechnology, 1:5000)]. Then the membrane was washed five times with TBST and once with TBS, each for 5 minutes. Chemiluminescence detection was performed using SuperSignal West Femto Maximum Sensitivity Substrate (Thermo, USA).” (Pages 15-16, Lines 292-325)

“**Histological analysis**: Hematoxylin and eosin (H&E) stained sections were microscopically re-evaluated to confirm the histological diagnosis of each sample. Paraffin-embedded tissue sections were dewaxed and rehydrated using xylene and a series of ethanol solutions with varying concentrations. After deparaffinization, sections were stained with hematoxylin for 5 minutes, rinsed in ddH2O, and then stained with eosin for 5 minutes. This was followed by dehydration in ethanol, clearing in xylene, and mounting with neutral balsam. Immunohistochemical (IHC) analysis was performed using the above tissue samples. After deparaffinization, antigen retrieval was carried out using Tris-EDTA buffer (Servicebio, pH 9.0). The sections were permeabilized with 0.1% Triton X-100 and blocked with 5% BSA to prevent nonspecific binding. Primary antibody was then applied, and the slides were incubated overnight at 4℃: anti-DNTTIP2 (ThermoFisher Scientific, TDIF2 Polyclonal Antibody, PA5-60731, 1:500), anti-MIF (Cell Signaling Technology, MIF (E7T1W) Rabbit mAb #87501, 1:400) and anti-CD74 (Servicebio, CD74 Rabbit pAb #GB115427, 1:1000). After washing with PBS, a labeled secondary antibody was added and incubated at room temperature for 60 minutes: Goat anti-Rabbit IgG-HRP Antibody (Absin, 1:500). Following another round of washing, DAB substrate (Servicebio) was added for color development, and the staining was monitored under a microscope to determine the optimal stopping time. The sections were then counterstained with hematoxylin (Servicebio) for 2 minutes and rinsed with running water. Finally, the slides were dehydrated through graded ethanol, cleared in xylene until transparent, mounted with neutral balsam, and observed under a light microscope.” (Pages 18-19, Lines 348-373)

**Q2: *There are several grammatical issues throughout the manuscript, including some abbreviations that are repeated upon first use, which may create confusion for readers.***

**Response:** Thank you for pointing out the grammatical issues and concerns regarding abbreviations. We have carefully reviewed the entire manuscript to address these points, ensuring that all abbreviations are introduced correctly and consistently, and that grammatical errors have been corrected. We believe these revisions enhance the clarity and readability of the manuscript. For example:

“Through transcriptome sequencing of our HBx-expressing HepG2 cells and analysis of HCC patient data from the cancer genome atlas (TCGA) and genotype-tissue expression (GTEx), we identified seven HBx-related genes, nuclear VCP-like (NVL), WD repeat domain 75 (WDR75), NOP58 nucleolar protein (NOP58), Brix domain-containing protein 1 (BRIX1), deoxynucleotidyltransferase terminal interacting protein 2 (DNTTIP2), MKI67 FHA domain interacting nucleolar phosphoprotein (NIFK), and ribosome production factor 2 (RPF2), associated with poor prognosis.” (Page 2, Lines 28-36)

“Rawal et al. have found that HBx-positive liver cancer cells exhibited increased migration and invasion capabilities via endothelial-derived transforming growth factor-β (TGF-β) and facilitated epithelial-mesenchymal transition (EMT) through cluster of differentiation (CD) 133 expression, thereby enhancing tumor aggressiveness[16]. ” (Page 5, Lines 88-93)

“However, in patients with chronic HBV infection, the functionality of these cells is compromised, characterized by limited proliferation and increased expression of inhibitory receptors such as programmed cell death protein 1‌ (PD1) and cytotoxic T lymphocyte-associated antigen 4 (CTLA4) [18-20].” (Page 5, Lines 102-106)

**Q3: *Although the study mentions the selection of seven hub genes, it states that "the expression levels of these seven genes were significantly upregulated in HCC cells expressing HBx and correlated with poor prognosis in HCC patients." Is there a potential bias in this observation that needs to be addressed?***

**Response:** Thank you for your thoughtful comment. To reduce potential bias, we performed LASSO regression analysis on the seven initially identified hub genes. This method helps identify the most important predictive variables by shrinking the coefficients of less relevant features to zero. As a result, only four key hub genes were retained for further analysis, ensuring a more robust and reliable selection process.

**Q4: *The authors' description of the methodology for selecting model genes is somewhat vague, and the graphical results are not clearly presented, which may hinder the interpretation of the findings.***

**Response:** Thank you for your comment. We appreciate your concern regarding the clarity of our methodology and results. In our revised manuscript, we have made efforts to clearly present the rationale and process behind the selection of model genes. Specifically, we used LASSO Cox regression to identify the most prognostically relevant genes among the seven hub genes, ultimately narrowing the model down to four key genes. The results are supported by both graphical representations and subsequent experimental validation, which we believe strengthens the reliability and interpretability of our findings.

We have made several revisions to improve clarity in the manuscript, for example:

“All identified DEGs met the criteria of P < 0.05 and |log_2_ (Fold-change)| > 0.5.” (Page 9, Lines 189-190)

“**Fig 2.** **E-G** The enriched GO terms of DEGs: (E) Biological Process, (F) Cellular Component, and (G) Molecular Function. Red and blue dots represent upregulated and downregulated genes based on log_2_FC. Inner box color indicates pathway z-score, and box size reflects enrichment significance.”

“**Fig 3.** **F** Colors represent different ranges of Pearson correlation coefficients (e.g., dark red indicates R = 0.8 - 1.0). The colored areas are categorized based on correlation strength, not proportionally scaled to the R value.”

“**Fig 4.** **C** The optimal cut-off point to divide riskscore into low and high groups was 5.39294. The height of each column in the histogram represents the number of patients within the corresponding riskscore range.”

“**Fig 6. A** Differences in TME infiltration cells between high-risk and low-risk groups. Enrichment scores represent the relative abundance of immune cell infiltration; higher scores indicate greater infiltration levels.”

“The correlation between the riskscore and immune infiltrating cells was illustrated (Fig 6B), showing varying degrees of association. It further illustrates the relationships between the risk score and key genes involved in immunotherapy and targeted therapy (Fig 6C).” (Pages 24-25, Lines 490-494)

“Among the seven prognosis-related genes, four genes with non-zero coefficients were retained by LASSO regression for constructing the riskscore model, while the remaining three were excluded due to minimal predictive contribution.” (Page 22, Lines 436-439)

“The result of qPCR and Western blot analysis indicated that DNTTIP2 mRNA and protein expression was significantly upregulated in HepG2 cells expressing HBx (Fig 8C, D). Moreover, we examined the expression of MIF and CD74, key molecules in the MIF signaling pathway, and found that their protein levels were also elevated in HBx-overexpressing HepG2 cells (Fig 8D). Furthermore, HCC tissue samples were collected from both HBV-positive and HBV-negative patients, and H&E staining was used to confirm the samples as HCC tissues (Fig 8E). DNTTIP2, MIF and CD74 expression levels in HBV-positive HCC tissues, marked by brown staining, exceeded those in HBV-negative HCC tissues (Fig 8E). ” (Pages 27-28, Lines 549-559)

**Fig 8.** Correlation of DNTTIP2 with MIF and verification of DNTTIP2, MIF and CD74 expression in HBV-related hepatocellular carcinoma.

**A** Gene expression levels of DNTTIP2 and MIF in GSE202642 dataset. **B** Correlation between DNTTIP2 and MIF expression based on TCGA cohort. **C** Expression of DNTTIP2 mRNA in HepG2 cells transfected with HBx plasmid or control plasmid (NC). **D** Expression of DNTTIP2, CD74 and MIF protein after transfection with HBx plasmid and control plasmid (NC) in HepG2 cells. **E** Representative images of H&E staining and IHC staining of HBV-negative and HBV-positive HCC tissue. IHC staining was performed to detect DNTTIP2, MIF, and CD74 expression. Brown staining indicates positive expression. Scale bars, 100 μm and 20 μm. Data presented as mean ± SEM (n = 3). *P < 0.05; **P < 0.01; ***P < 0.001; ****P < 0.0001.

**S1 Fig.** Characterization of HBx-Related DEGs and MIF Signaling Interactions in HBV-Related HCC.

**A** PPI network of HBx-related DEGs. **B** The number of interactions and interaction strength between different celltypes in HBV-related HCC tissues. **C** The interaction strength of MIF signaling pathway between different celltypes in HBV-related HCC tissues. **D** Signaling from malignant cells to other cells in the MIF signaling pathway. A thicker line indicates a higher interaction strength or a greater number of interactions.

**Q5: *The study reference transcription factors and miRNAs, presenting relevant results; however, the authors do not discuss the significance of this portion of the results in the analysis.***

**Response:** Thank you for your insightful comment. After careful evaluation, we recognized that the transcription factor and miRNA analysis, while informative, had limited relevance to the core focus of our study. Therefore, we have removed the corresponding content, specifically Figure 3G and the related description in the Results and Methods section, to ensure a clearer and more focused presentation of the manuscript.
